# Quantum Chemical Studies on the Structural, Electronic, and Vibrational Properties of Boron Carbonitride Nanotubes

**DOI:** 10.1021/acsomega.4c09158

**Published:** 2025-04-17

**Authors:** Raúl Mendoza-Báez, Dolores Garcia-Toral, María Teresa Romero de la Cruz, Aracely del Carmen Martínez Olguín, Víctor M. Vázquez-Báez, Gregorio Hernández Cocoletzi, Juan Francisco Rivas-Silva

**Affiliations:** † Departamento de Química, Centro de Investigación y de Estudios Avanzados del IPN (Cinvestav), Av. IPN 2508, Col. San pedro Zacatenco, Ciudad de México 07360, Mexico; ‡ Benemérita Universidad Autónoma de Puebla, Facultad de Ingeniería Química, Av. San Claudio y 18 Sur S/N, San Manuel, Puebla 72570, Mexico; § Facultad de Ciencias Físico Matemáticas, Universidad Autónoma de Coahuila, Unidad Camporredondo, Edif. A, 25000 Saltillo, Coahuila, Mexico; ∥ CONAHCyT–Facultad de Ciencias Físico Matemáticas, Universidad Autónoma de Coahuila, Unidad Camporredondo, Edif. A, 25000 Saltillo, Coahuila, Mexico; ⊥ Facultad de Ingeniería, Benemérita Universidad Autónoma de Puebla, Puebla 72570, Mexico; # Instituto de Física, Benemérita Universidad Autónoma de Puebla, Av. San Claudio y Blvd. 18 Sur, Col. San Manuel, Puebla 72570, Mexico

## Abstract

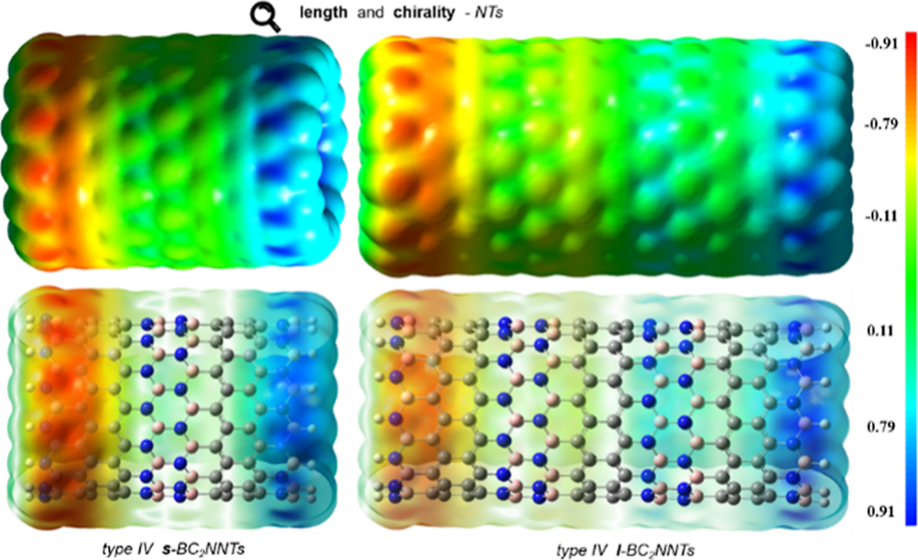

The structural, vibrational, and electronic properties of zigzag (***n***, 0) BC_2_N nanotubes are investigated in their most stable configuration, type IV. Studies are based on density functional theory (DFT) using the M06-2X/6-31G(d) level of theory. The property–structure relationship is investigated by focusing on the chirality index (***n***). Furthermore, to analyze the length dependence of the stability/reactivity of BC_2_N nanotubes, short (***n*** = 5–14, ***s***-BC_**2**_NNTs) and long (***n*** = 5–13, ***l***-BC_**2**_NNTs) nanotubes were proposed, with average lengths of 18.07 and 26.74 Å, respectively. Total energy minimization, assuming nonmagnetic nature and charge neutrality, yielded the ground state of all nanostructures. Results show that the electrophilicity and nucleophilicity indices exhibit that the BC_2_NNTs are electrophilic systems; however, an increase in the length of the nanotube triples its electrophilic character. The ***s***-BC_2_NNTs show a semiconductor character, while ***l***-BC_2_NNTs show a semiconductor-to-semimetallic character; therefore, the length of the nanotube is a key element for fine-tuning the conductive properties of these systems. Nanotubes of larger length and diameter are favored, based on analysis of cohesion energies. Furthermore, a longer axial length of the nanotube improves the solubility properties as it considerably increases the dipole moment and the solvation energy in water. Finally, BC_2_NNTs showed polarization relative to the distribution of negative and positive charges, as indicated by molecular electrostatic potential maps. This is important for possible regioselective reactions. The set of BC_2_NNTs studied in this work may be proposed for biological applications. Also, due to the molecular gap energy found in the range 0.35 < *E*_g_ < 1.6 eV, we propose that these structures could be applied in the fabrication of integrated circuits at the nanoscale.

## 1 Introduction

**Figure 1:**
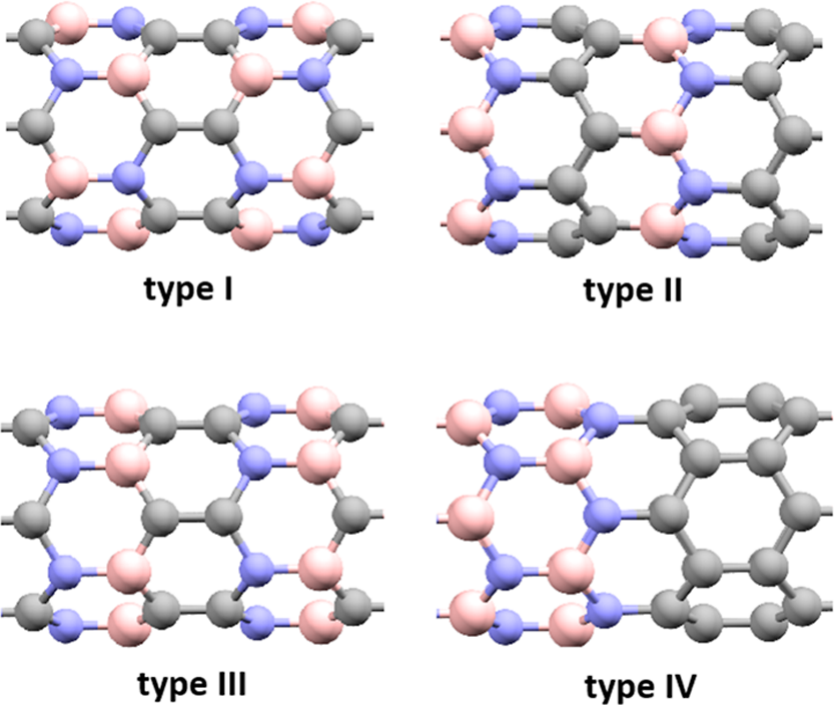
Different types of structural models of zigzag-BC_2_N nanotubes. (Nitrogen, boron, and carbon atoms in blue, pink, and gray balls, respectively).

One-dimensional (1-D) nanomaterials have become the focus of attention within the field of nanotechnology due to their wide number of applications such as high-performance memories, sensors, nanoelectronics, optoelectronics, hydrogen storage, among others, as well as the diversity of different nanostructures.^1−4^ Without a doubt, nanotubes are among the most studied 1-D nanomaterials. Carbon nanotubes (CNTs), discovered in 1991 by Iijima,^5^ and boron nitride nanotubes (BNNTs), synthesized in 1995 by Chopra,^6^ are the main exponents within this type of nanostructures. Despite the structural similarity between these nanotubes, the electronic properties of CNTs are dependent on chirality,^7−10^ while BNNTs, and other inorganic nanotubes such as BPNTs and SiCNTs, show to have chirality-independent electronic properties when the diameters are large.^11−15^ CNTs can be metallic or semiconductors, while BNNTs are electrical insulators.^16^ However, BNNTs are more thermally stable and better resist oxidation.^17,18^ This inspired the development of new hybrid carbon–boron nitride materials with the aim of achieving physical properties intermediate to those of the precursors, resulting in the so-called ternary BCN materials.^19,20^ Various materials have been synthesized with different proportions of elements B, C and N, forming a range of boron carbonitride (B_*x*_C_*y*_N_*z*_) compounds with diverse applications in optics, electronics and as gas adsorbers.^21−23^ Within the BCN family, BC_2_N exhibits one of the most stable stoichiometry and has attracted extensive research attention.^24−26^ In this sense, BC_2_N nanotubes (BC_2_NNT) were synthesized in the early 90s and,^27,28^ since then, have shown potential applications such as electronic devices, hydrogen storage, O_2_ sensors and energy materials.^29−37^ Theoretical studies show that BC_2_NNT nanotubes serve as adsorbers for molecules such as glycine, tryptophan, fluorouracil, carbon monoxide and hydrogen cyanide, as well as chloride, fluoride and sodium ions.^38−43^ Different structural arrangements have been proposed for BC_2_N monolayers, differentiated by the periodic distribution of B, C and N atoms throughout the structure.^28,44−48^ The reported band gap values are in a range of 0.0 to 1.62 eV. Previous first-principles studies have shown that by maximizing the number of C–C and B–N bonds, and minimizing unwanted mononuclear B–B and N–N bonds, a more energetically stable structure is obtained. The C–N and C–B bonds should also be avoided because they increase structural stress. That is, the desire is to increase the number of higher energy bonds (B–N > C–C > N–C > B–C > B–B > N–N).^48^ Consequently, these characteristics also apply to the BC_2_N nanotubes formed by said monolayers. Figure 1 shows the four main types of BC_2_NNTs (zigzag chirality) reported in the literature. Type-IV and type-I configurations are the most and least stable, respectively (IV > II > III > I).^46,49^

Different groups have focused their interest in studying the electronic properties of these systems and their relationship to structural properties. For example, H. Pan et al.^50,51^ showed that the electronic properties of type-II BC_2_N nanotubes are closely related to both the diameter and chirality of the tube. A few years later, Zahedi described the semiconductor character of type-III BC_2_NNTs and its dependence on size and chirality.^52^ Recently, Majidi and Ayesh^47^ studied an interesting isomer of BC_2_N nanotube formed from T-graphene sheets, called T-BC_2_NNT, which possess both metallic and semiconducting properties. However, we consider that it is important to pay special attention to those geometries with greater stability, that is, type-IV BC_2_NNTs. Furthermore, type-IV BC_2_N nanotubes are mostly direct band semiconductors, unlike type-II and -III nanotubes that have an indirect band for some chiralities.^49,52^ Azevedo et al.^53,54^ were the first to study the electronic-structural relationship in type IV BC_2_NNTs, finding that the energy gap is affected by the diameter and curvature of the tube. Then, Akhavan et al.^49^ reported the electronic and structural properties for type-IV BC_2_NNTs in zigzag and armchair chirality, where the nanotubes have a metallic-to-semiconducting character, showing that the diameter and chirality significantly impacts the band gap energy of zigzag-type nanotubes. The main contribution of the work is the electronic-structural relationship of type-IV BC_2_NNTs where the length of the nanotubes is considered as a dependent parameter, their reactivity is described, and finally, IR spectra are considered as a tool for the characterization of these systems.

In this work, the structural, electronic, and vibrational properties of zigzag type-IV (*n*,0) BC_2_NNTs (*n* = 5–14) were studied through calculations based on density functional theory (DFT) to perform the finite molecular study. A complete characterization of the stability and reactivity of these systems was carried out by means of global molecular descriptors: electrophilicity (ω) and nucleophilicity (*N*″) indices, global hardness (η) and chemical potential (μ). As well as dipole moment, solvation energy, cohesive energy, and MEP isosurface (electrostatic potential maps). A better understanding of the nature of type-IV BC_2_N nanotubes will enhance their uses and applications in future technological developments.

## 2 Theoretical Methods

First-principles calculations were performed, using Density Functional Theory (DFT), for type-IV zigzag (*n*,0) BC_2_N nanotubes (with *n* = 5–14). To perform the finite molecular study of BC_2_NNTs. We propose nanotubes with two different lengths: short ones (*s*-BC_2_NNT) with one six-membered ring belt of boron-nitride and two six-membered ring belts of carbon atoms, while the long ones (*l*-BC_2_NNT) have two belts of BN and three belts of carbon. In both cases, the ends of the nanotubes are single chains of boron nitride passivated with hydrogen atoms (Figure S1, Supporting Information). There is a relationship between the number of atoms of each chemical species that constitute the nanotubes and the chirality index (*n*), where *H* = 2*n*, *C* = 8*n*, and *B* = *N* = 4*n* for the *s*-BC_2_NNT, while for the *l*-BC_2_NNT, *H* = 2*n*, *C* = 12*n*, and *B* = *N* = 6*n*. Nanotubes were optimized with a singlet multiplicity (*M* = 1) and a neutral charge (*Q* = 0). Vibrational frequency calculations were carried out to evaluate structural stability, verifying that they relax with local minima and showing that all nanotubes have nonimaginary frequencies. The calculations consider gas phase systems within the M06–2X/6–31G(d) approach implemented in the Gaussian 16 code.^55^ The global-hybrid functional M06–2X has been shown to have better performance compared to local functionals in calculations of binding (dissociation) energies, reaction and activation energies, transition states, electronic excitation energies, and radicals.^56−59^ Furthermore, the M06–2X functional is suitable for medium-sized systems and for unraveling noncovalent interactions.^60−62^

The reactivity of nanotubes can be studied through the global molecular descriptors, which are calculated from the energies of the frontier molecular orbitals HOMO (highest occupied molecular orbital) and LUMO (lowest unoccupied molecular orbital), as established by the Koopmans theorem,^63^ where *I* = −*E*_HOMO_ and *A* = -*E*_LUMO_ (*I* is the ionization potential and *A* is the electronic affinity). Therefore, the chemical potential (μ) and global hardness (η) are given by the following equations
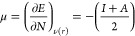
1
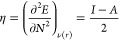
2where *E* is the total energy, *N* is the number of electrons, and ν(*r*) is the external potential of the system.^64^ Based on these, other molecular descriptors can be defined such as the electrophilicity index (ω) and the nucleophilicity index (*N*^″^) (eqs 3 and 4, respectively).
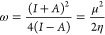
3
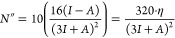
4

The electrophilicity index measures the ability of a molecule to accept electrons in an electron-rich environment.^65,66^ A good electrophile is characterized by high values of ω, that is, high μ values and low η values. The nucleophilicity index was proposed as an inverse relationship to the electrodonating power (ω^–^),^67,68^ although it can also be directly related to the global hardness, as in eq 4. This descriptor has recently been used to describe the relative nucleophilicity in inorganic nanotubes.^69^ On the other hand, to evaluate the stability of the nanotubes, the cohesive energy (*E*_coh_) was calculated, which is defined as the energy necessary to break all the bonds, leaving the system with the individual components.^70−72^ The *E*_coh_ is obtained from the following equation
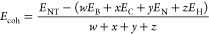
5where *E*_NT_ is the total energy of the nanotube, while *E*_B_, *E*_C_, *E*_N_, and *E*_H_ represent the total energies of the isolated boron, carbon, nitrogen, and hydrogen atoms, respectively. The coefficients *w*, *x*, *y*, and *z* indicate the number of atoms corresponding to each chemical species that constitutes the nanotube. *E*_coh_ is expressed in eV/atom; more stable systems will show more negative cohesive energy values.

## 3 Results and Discussion

### 3.1 Structural Properties

Tables S1 and S2 summarize the structural parameters of *s*-BC_2_NNT and *l*-BC_2_NNT, respectively. In the case of the *s*-BC_2_NNT, the average axial length is 18.07 Å. The large nanotubes are about 48% longer than the short ones, with an average axial length of 26.74 Å. In both cases, a negligible increase in the value of the axial length is observed as the chiral index (*n*) increases. At the ends of the optimized nanotubes, it is observed that the N–H bond length is shorter than the B–H bond, 1.017 and 1.180 Å, respectively, because the difference in electronegativity in the N–H pair is greater than that in B–H, generating a stronger attraction and, therefore, shorter N–H bonds. The bond lengths are very similar between the two types of nanotubes, as indicated in Table 1, showing that the increase in the axial length does not modify the intramolecular bond lengths. The bond distances can be ordered in descending order as follows: B–C > B–*N* > C–C > N–C > B–H > N–H. These bond distances are very close to those reported by Akhavan et al.^49^ for analogous nanotubes. Furthermore, it is worth mentioning that the average bond length of B–N (1.45 Å) and C–C (1.44 Å) bonds in the zigzag-type BC_2_NNT agrees well with what was reported for BNNTs and CNTs (zigzag-type), respectively.^73−75^ This suggests a partial double bond (sp^2^) nature for B–N and C–C bonds in the *s*(*l*)-BC_2_NNTs. Similarly, the B–C bond length (1.53 Å) suggests a (small) partial double bond character since experimental and theoretical evidence shows that this type of B–C bond presents bond lengths of 1.49 to 1.59 Å.^76−78^

**Table 1:** Comparison between the Average Bond Length Values (in Å) of ***s***-BC_2_NNT and ***l***-BC_2_NNTs

nanotube	average bond lengths (Å)					
zigzag	B–C	B–N	C–C	N–C	B–H	N–H
*s*-BC_2_NNT	1.537	1.451	1.428	1.405	1.181	1.016
*l*-BC_2_NNT	1.533	1.453	1.429	1.401	1.180	1.016

**Figure 2:**
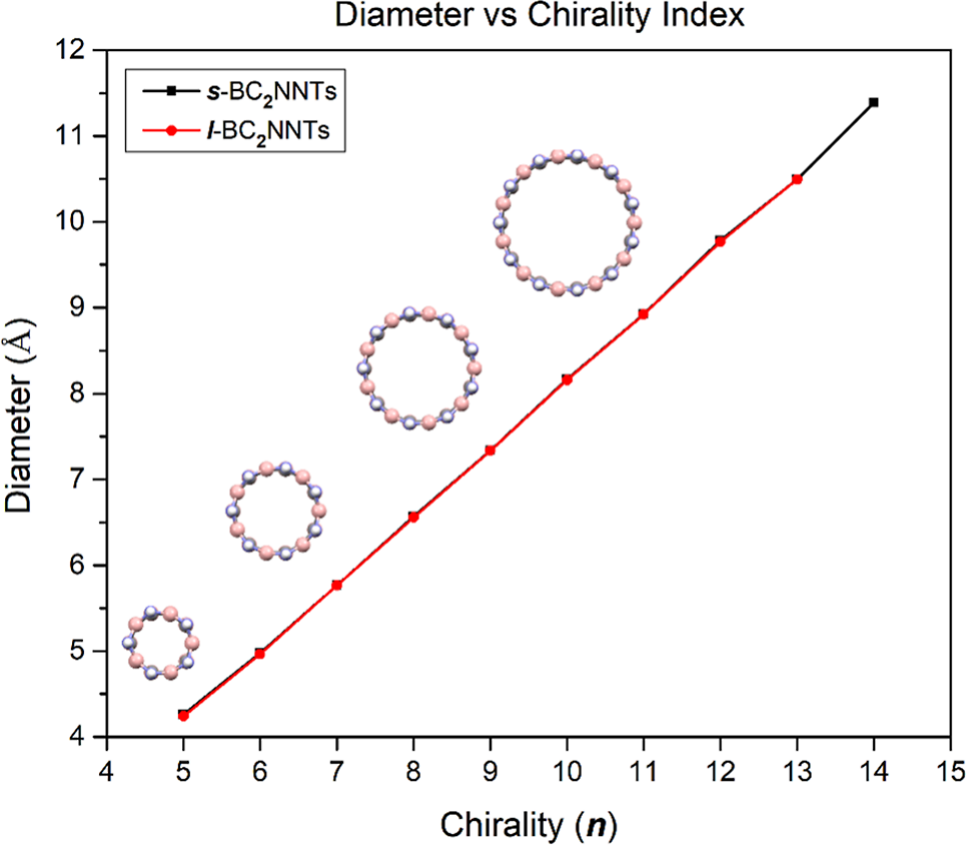
Nanotube diameter size (Å) as a function of the chirality index (***n***). Solid black and red lines for zigzag-type *s*-BC_2_NNTs and *l*-BC_2_NNTs, respectively.

There is a linear relationship (coefficient of determination, *R*^2^ = 0.999) between the size of the nanotube diameter and the chirality index, as shown in Figure 2. As the ***n*** value increases, the diameter of the nanotube lengthens. There is no significant difference in diameter sizes between two nanotubes with the same chirality index, regardless of whether the nanotube is *s*- or *l*-BC_2_NNT, which is why in Figure 2, the lines overlap.

According to the standard values of the literature on Mayer’s analysis, Table 2 shows the average values of the corresponding bonds for the type-IV BC_2_NNT systems treated here and which would be classified by the bond order.^79−81^

**Table 2:** Calculated Mayer Bond Order of the ***s***-BC_2_NNTs and ***l***-BC_2_NNTs

nanotube	mayer bond order					
zigzag	B–C	B–N	C–C	N–C	B–H	N–H
BC_2_NNT	1.067	1.116	1.378	1.077	0.962	0.855

The N–H and B–H bond order slightly less than 1 is typical for this type of single bonds in organic and inorganic compounds.^82^ The B–C, N–C, and B–N bonds have values slightly greater than 1, with a typical single bond nature, with B–N being the strongest. The homonuclear C–C bond has the highest Mayer’s bond order, whose value suggests a character closer to a partial double bond.

The dipole moment (μ⃗) of the molecules is the vector sum of the individual bond dipoles and allows us to define whether it is polar or nonpolar. It is well-known that zigzag-type nanotubes are polar systems, regardless of the chemical nature of their constituent atoms. Table 3 and Figure S2 show how the dipole moment increases as the chirality index increases. In this case, the length of the nanotube does considerably influence the μ⃗ values since the *l*-BC_2_NNTs have μ⃗ values between 1.7 and 2.1 times higher than the *s*-BC_2_NNTs. The high μ⃗ values of BC_2_NNTs are considerably higher than those reported for zigzag-type CNTs and BNNTs,^83−86^ indicating that BC_2_NNTs are more polar systems, which is very important for applications in biological and medical systems. However, tuning the dipole moment based on the nanotube length involves a synthetic challenge to selectively obtain nanotubes with a specific length.

**Table 3:** Dipole Moments (in Debye) of the Two Types of BC_2_NNT Proposed in This Work

nanotube	dipole Moment (debye)									
zigzag	(5,0)	(6,0)	(7,0)	(8,0)	(9,0)	(10,0)	(11,0)	(12,0)	(13,0)	(14,0)
*s*-BC_2_NNT	10.56	22.47	26.62	25.65	32.47	31.33	36.90	37.83	41.60	44.14
*l*-BC_2_NNT	18.23	46.29	56.00	54.98	60.32	67.24	66.54	75.06	77.11	

### 3.2 Electronic Properties

**Figure 3:**
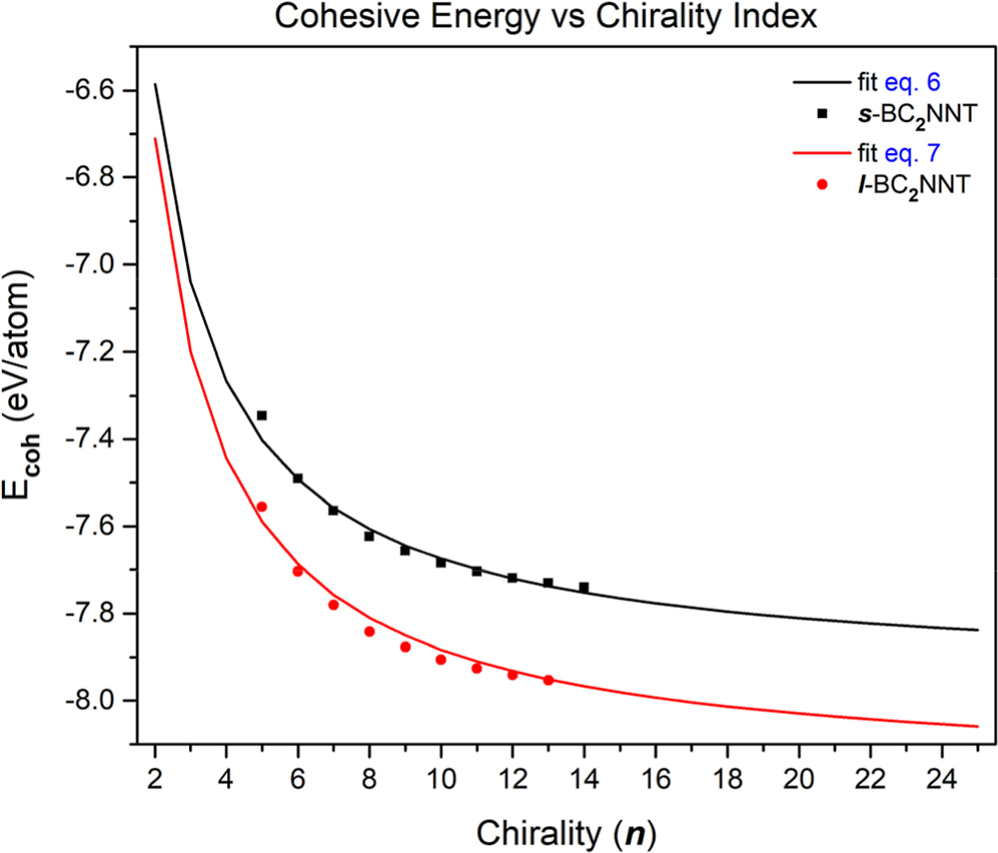
Cohesive energy, *E*_coh_ (in eV/atom) behavior as a function of chirality index (***n***). Solid black and red lines for fitting model equations for zigzag-type ***s***-BC_2_NNTs and ***l***-BC_2_NNTs, respectively.

Tables 4 and 5 summarize the electronic properties (frontier molecular orbitals, global molecular descriptors, and cohesive energy) of *s*-BC_2_NNT and *l*-BC_2_NNT, respectively. First, we will address the relationship between the total energy (*E*_NT_), the cohesion energy (*E*_coh_), and the chirality index (***n***). The *E*_NT_ of the nanotubes (both in ***s***-BC_2_NNT and in ***l***-BC_2_NNT) increases linearly as a function of the ***n*** index. Figure S3 shows the linear behavior and the equations of the linear fit, with *R*^2^ = 1.0. Furthermore, for the same ***n*** value, the ratio of *E*_NT_(***l***-BC_2_NNT) to *E*_NT_(***s***-BC_2_NNT) is equal to 1.5, that is, the total energy of ***l***-BC_2_NNT is about 50% higher compared to that of ***s***-BC_2_NNT. This agrees well with the 48% increase in nanotube length when going from ***s***- to ***l***-BC_2_NNT. If the total energy can be defined as a function of ***n***, and this is a parameter to calculate the cohesive energy, therefore, we can also define *E*_coh_ as a function of ***n*** index (eqs 6 and 7 for ***s***- and ***l***-BC_2_NNTs, respectively).
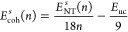
6
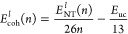
7In the first term: *E*_NT_^*s*,*l*^ is the function of the total energy resulting from the linear fit, while 18*n* and 26*n* are the number of atoms that constitute the ***s***- and ***l***-BC_2_NNTs, respectively. In the second term: *E*_uc_ is the total energy of the unit cell (as defined in refs (46) and (49) for type-IV BC_2_NNTs, see Figure S4), which is a constant value independent of ***n***, and denominator 9 is the number of atoms that make up the said unit cell. That is, the first and second terms represent the average energy per atom of the entire nanotube and the unit cell, respectively. Figure 3 shows the behavior of *E*_coh_ as a function of the chirality index, fitting the data obtained through computational calculations to the model expressed in eqs 6 and 7. It is noted that the cohesive energy for *l*-BC_2_NNTs is slightly higher, indicating that a considerable increase in the length of the nanotube (48%) improves the stability by 0.2 eV/atom on average (∼2.7%). The cohesive energy of both types of nanotubes is between 6.6 and 8.2 eV/atom for a range of ***n*** = 2–25, with a substantial increase in stability as the diameter of the nanotube grows. This result confirms that type-IV BC_2_NNTs nanotubes would be more stable than other BC_2_NNT isomers (type I, II, III, and others) since the latter have *E*_coh_ values lower than 5.2 eV/atom.^44,48^ Additionally, compared to other inorganic nanotubes, ***s***- and ***l***-BC_2_NNTs (average *E*_coh_: −7.73 eV/atom) are as stable as BNNTs (−7.27 eV/atom) and more stable than BPNTs (−2.95 eV/atom).^15,87^ At large diameters (or high ***n*** values), all nanotubes will have almost equal energies, showing that the stability of large diameter BC_2_NNTs will not depend on chirality.

**Table 4:** Optimized Total Energy (*E*_NT_), Energy of the Frontier Molecular Orbitals (HOMO and LUMO), Molecular Gap Energy (*E*_g_), Global Molecular Descriptors (η,μ,ω,*N*″), and Cohesive Energy (*E*_coh_) for the (*n*,0) *s*-BC_2_NNTs (*n* = 5–14)^a^

zigzag (*n*,0)	global molecular descriptors for *s*-BC_2_NNTs									
descriptors	(5,0)	(6,0)	(7,0)	(8,0)	(9,0)	(10,0)	(11,0)	(12,0)	(13,0)	(14,0)
*E*_NT_^b^	–84.9410	–101.9448	–118.9449	–135.9455	–152.9441	–169.9429	–186.9409	–203.9388	–220.9364	–237.9338
*E*_HOMO_	–4.5448	–4.1292	–4.1635	–4.1932	–4.0903	–4.1937	–4.1020	–4.1874	–4.1219	–4.1798
*E*_LUMO_	–3.5578	–2.8963	–2.7303	–2.5922	–2.9284	–2.7377	–2.9779	–2.9477	–2.9904	–3.0543
*E*_g_gap	0.9871	1.2330	1.4332	1.6010	1.1620	1.4560	1.1242	1.2398	1.1315	1.1255
*I* = −*E*_HOMO_	4.5448	4.1292	4.1635	4.1932	4.0903	4.1937	4.1020	4.1874	4.1219	4.1798
*A* = −*E*_LUMO_	3.5578	2.8963	2.7303	2.5922	2.9284	2.7377	2.9779	2.9477	2.9904	3.0543
η=(*I* – *A*)/2	0.4935	0.6165	0.7166	0.8005	0.5810	0.7280	0.5621	0.6199	0.5658	0.5628
μ = −(*I* + *A*)/2	–4.0513	–3.5127	–3.4469	–3.3927	–3.5093	–3.4657	–3.5399	–3.5676	–3.5561	–3.6171
ω = μ^2^/2η	16.6278	10.0078	8.2902	7.1894	10.5987	8.2492	11.1470	10.2659	11.1762	11.6239
*N*^″^	0.5343	0.8445	0.9898	1.1129	0.8048	0.9927	0.7700	0.8246	0.7678	0.7406
*E*_coh_	–7.3440	–7.4877	–7.5617	–7.6206	–7.6541	–7.6821	–7.7009	–7.7159	–7.7275	–7.7365

a Optimization was performed via DFT/M06-2*X*/6-31G(d).

b Total energy is in units of keV; the rest of the parameters are given in eV.

**Table 5:** Optimized Total Energy (*E*_NT_), Energy of the Frontier Molecular Orbitals (HOMO and LUMO), Molecular Gap Energy (*E*_g_), Global Molecular Descriptors (η,μ,ω,*N*″), and Cohesive Energy (*E*_coh_) for the (*n*,0) *l*-BC_2_NNTs (*n* = 5–13)^a^

zigzag (*n*,0)	global molecular descriptors for *l*-BC_2_NNTs								
descriptors	(5,0)	(6,0)	(7,0)	(8,0)	(9,0)	(10,0)	(11,0)	(12,0)	(13,0)
*E*_NT_^b^	–127.3343	–152.8243	–178.3091	–203.7944	–229.2770	–254.7597	–280.2413	–305.7226	–331.2036
*E*_HOMO_	–4.4015	–3.7960	–3.7558	–3.8208	–3.7536	–3.7824	–3.7803	–3.8371	–3.7933
*E*_LUMO_	–3.9266	–3.3913	–3.2564	–3.0750	–3.3788	–3.2991	–3.4136	–3.4843	–3.4302
*E*_g_gap	0.4749	0.4047	0.4994	0.7458	0.3748	0.4833	0.3667	0.3528	0.3631
*I* = −*E*_HOMO_	4.4015	3.7960	3.7558	3.8208	3.7536	3.7824	3.7803	3.8371	3.7933
*A* = −*E*_LUMO_	3.9266	3.3913	3.2564	3.0750	3.3788	3.2991	3.4136	3.4843	3.4302
η=(*I* – *A*)/2	0.2375	0.2024	0.2497	0.3729	0.1874	0.2417	0.1833	0.1764	0.1816
μ = −(*I* + *A*)/2	–4.1640	–3.5937	–3.5061	–3.4479	–3.5662	–3.5408	–3.5969	–3.6607	–3.6118
ω = μ^2^/2η	36.5105	31.9083	24.6151	15.9392	33.9306	25.9380	35.2862	37.9859	35.9241
*N*^″^	0.2589	0.2965	0.3788	0.5647	0.2798	0.3605	0.2695	0.2510	0.2649
*E*_coh_	–7.5523	–7.7006	–7.7779	–7.8385	–7.8738	–7.9025	–7.9223	–7.9377	–7.9498

a Optimization was performed via DFT/M06-2*X*/6-31G(d).

b Total energy is in units of keV; the rest of the parameters are given in eV.

The electrophilicity (ω) and nucleophilicity (*N*″) indices show that the BC_2_NNTs are electrophilic systems, with the (5, 0) and (12, 0) nanotubes being the most electrophilic within the ***s***- and ***l***-BC2NNTs, respectively. The increase in nanotube length triples (on average) the ω values; therefore, ***l***-BC_2_NNTs are considerably more electrophilic compared to ***s***-BC_2_NNTs. This can be associated with the decrease in the energy (more negative) of LUMO orbitals when going from short (***s***-) to large (***l***-) nanotubes, making the LUMO orbitals more accessible, as seen in Figure S5. Concerning the chemical potential (μ), it shows an abrupt decrease in the μ energy (∼0.5 eV) when going from ***n*** = 5 to ***n*** = 6. When ***n*** ≥ 6, it is observed that the μ energy stabilizes in a narrow range of ∼ ± 0.2 eV. Interestingly, the length of the nanotube practically does not modify its chemical potential (Figure S6), with an increase of only ∼2% occurring.

**Figure 4:**
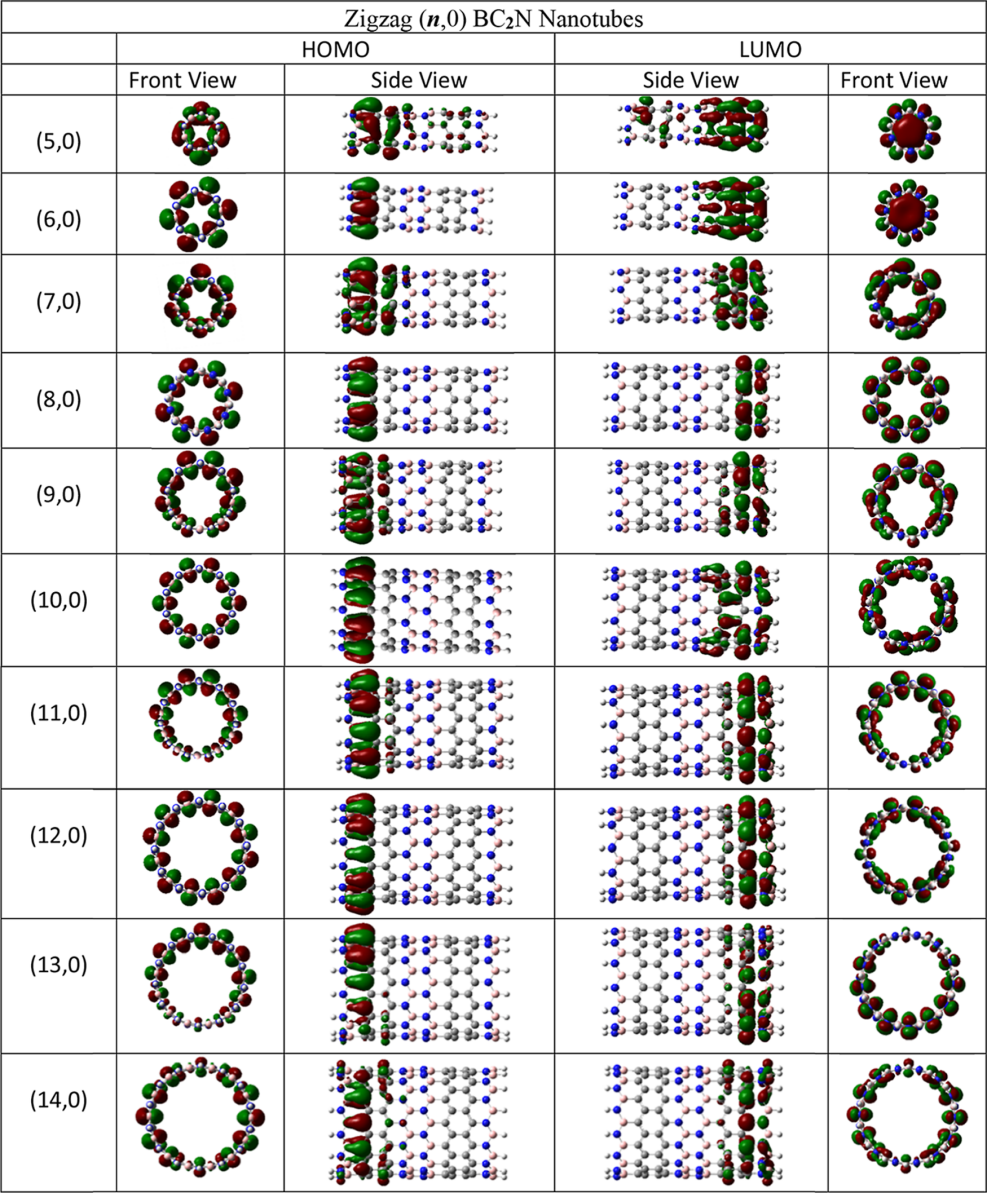
Spatial distribution of the HOMO and LUMO orbitals through the zigzag-type ***s***-BC_**2**_N nanotubes. Atom colors: pink, boron; blue, nitrogen; and gray, carbon. Calculated at DFT/M06-2X/6-31G(d).

**Figure 5:**
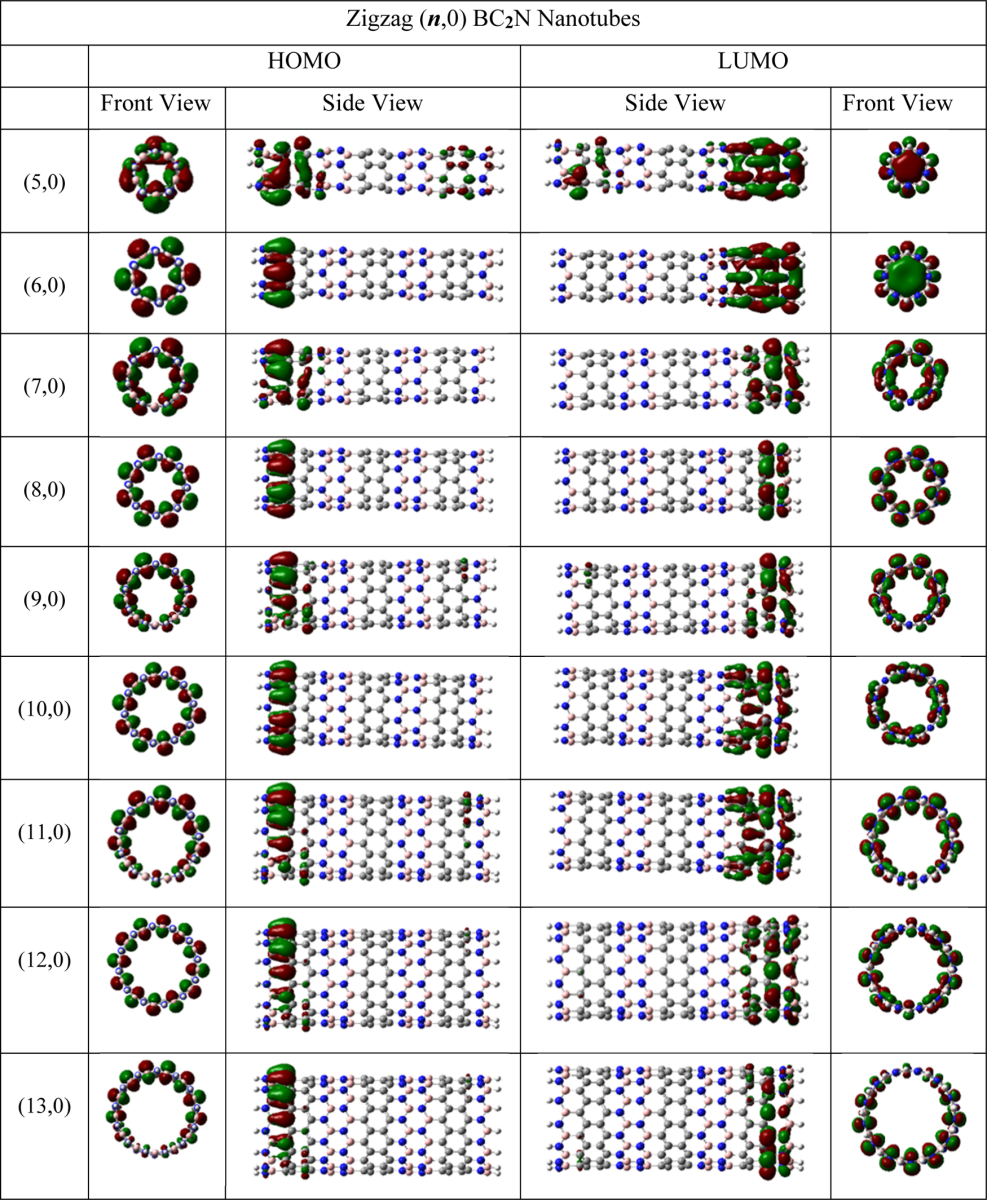
Spatial distribution of the HOMO and LUMO orbitals through the zigzag-type ***l***-BC_**2**_N nanotubes. Atom colors: pink, boron; blue, nitrogen; and gray, carbon. Calculated at DFT/M06–2*X*/6–31G(d).

The density distributions of HOMO and LUMO molecular orbitals are shown in Figures 4 and 5. The FMOs shape and how they are distributed along the nanotubes is quite similar in both cases. The HOMO is located almost exclusively at the N–H bonded end of the nanotube, called the nitrogen side. On the other hand, LUMO predominates at the opposite end, that is, on the boron side. However, when ***n*** = 5, it is observed that there is orbital density on both the N- and B-sides, suggesting that small diameters allow the HOMO and LUMO orbitals to permeate at both ends of the nanotube. Therefore, BC_2_NNT systems with controlled reactivity sites should have large diameters. The front view of the LUMO shows us that the smaller diameter BC_2_NNTs (***n*** = 5, 6) have orbital density in the form of a “cap” located in the cavity of the nanotube. When ***n*** > 6, the diameter is large enough to not allow this voluminous LUMO density. The HOMO orbitals are housed mainly on B–C bonds close to the N-side, whose shape suggests π-character of the orbital. For nanotubes (5,0) and (7,0), localized electron density is observed on C–C bonds contributed by π-orbitals. Furthermore, in the nanotubes (***n***, 0) where ***n*** = 5, 7, 9, 11, 13, and 14 in ***s***-BC_2_NNTs and (***n***, 0) where ***n*** = 5, 7, 9, 11, 12, and 13 in ***l***-BC_2_NNTs, there is a contribution of p orbitals located mainly on C atoms and, in some cases, also on N atoms. This observation was also described by Akhavan et al.^46^ when studying the band structure of some BC_2_NNTs, showing agreement in the analysis of the electron density distribution of BC_2_NNTs systems derived from finite-molecular studies (as in this work) or from periodic calculations of crystalline systems. Figure S7 shows the dependence of the molecular gap on the chirality index. The ***s***-BC_2_NNTs show a semiconductor character (1.60–0.98 eV), while ***l***-BC_2_NNTs show a semiconductor-to-semimetallic character (0.74–0.35 eV). This result suggests that although the HOMO–LUMO molecular gap (*E*_g_) depends on chirality, the length of the nanotube is a key element for fine-tuning the conductive properties of these systems. It should be noted that, in both ***s***- and ***l***-BC_2_NNTs, the maximum peaks in the *E*_g_ vs ***n*** index graph (Figure S7) correspond to those nanotubes that only present π-orbitals contributions on B–C bonds in the HOMO, that is, the nanotubes (8, 0), (10, 0), and (12, 0) for ***s***-BC_2_NNTs and (8, 0) and (10, 0) for ***l***-BC_2_NNTs. It seems that the fluctuations of the molecular gap converge to a constant value when the nanotubes have a large diameter (approximately ***n*** > 12); however, it is necessary to study more BC_2_NNTs at wider diameters. As in the HOMO–LUMO gap, the global hardness (η) decreases when going from ***s***- to ***l***-BC_2_NNTs, with the nanotube (8, 0) having the highest η values in both cases (0.80 and 0.37 eV, respectively). Systems with low hardness (or high softness) have high polarizability values and vice versa. Polarizability (α) is a measure of the responsiveness of a molecule to an electric field. Furthermore, large HOMO–LUMO gaps (*E*_g_) are associated with low polarizability due to the relationship between hardness (η) and the molecular gap: 2η = *E*_g_.

### 3.3 Vibrational Analysis

**Figure 6:**
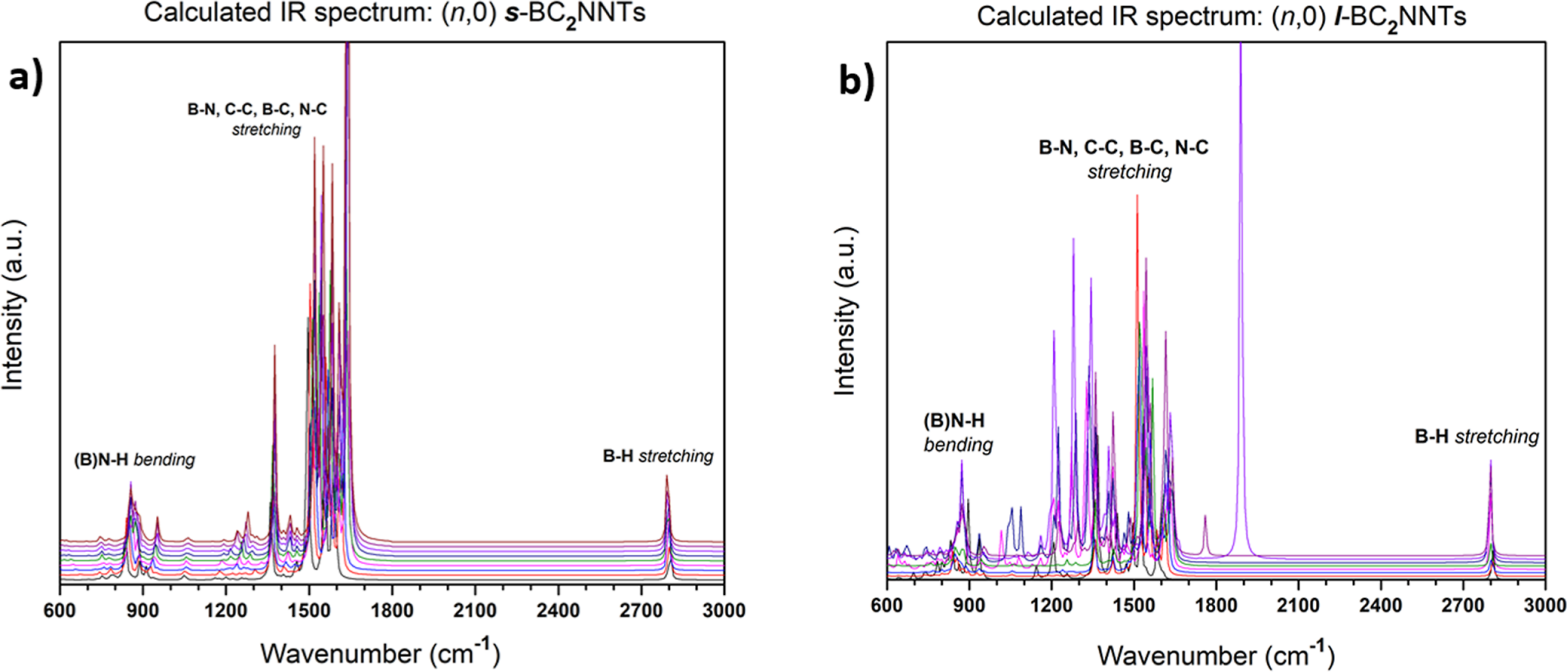
(a) Theoretical IR spectra of ***s***-BC_2_NNTs and (b) ***l***-BC_2_NNTs, both from 600 cm^–1^ to 3000 cm^–1^. The signal of the peaks was shifted by 700 ε units relative to each other. Lines from bottom to top correspond to ascending ***n*** values, respectively.

Figure 6a,b shows the calculated IR spectra of ***s***- and ***l***-BC_2_NNTs, respectively. No imaginary frequencies were found, indicating that all nanotubes are at global minima. In both cases, a pair of characteristic peak sets corresponding to vibrations located at the ends of the nanotubes are distinguished. The harmonic scaling factor of M06-2*X*/6-31G(d) (λ^H^ = 0.9827) was used to obtain more accurate values in the frequencies, which was proposed by Kesharwani et al.^88^ First, a strong wagging vibration (out-of-plane bending) generated by the B–N–H triad on the N-side of nanotubes was found in the 800–900 cm^–1^ region. The second set of peaks is located at higher wavenumbers, around 2800 cm^–1^, corresponding to a strong stretching of the B–H bond on the B-side. It seems that these characteristic peaks are independent of the BC_2_N nanotube length. This is not the case for the stretching vibrations of the B–N, C–C, B–C, and N–C bonds along the nanotube axis, which are modified by the influence of the length of the nanotube. For ***s***-BC_2_NNTs, these stretching vibrations occurs mainly in the 1500–1700 cm^–1^ range, with these peaks almost overlapping (Figure 6a). However, for ***l***-BC_2_NNTs, there is a greater number of relatively high intensity peaks in a much wider range of wavenumbers, from 900 to 2000 cm^–1^. Thus, longer nanotubes allow a greater number of stretching vibrations in homo- and heteroatomic bonds along their axis. Experimentally obtained IR spectra of boron–carbonitride nanotubes reported in the literature describe the following: broad absorption peaks at 784 cm^–1^ corresponding to out-of-plane bending of the B–N–B bond, close to our values obtained for the same vibrational mode but which we assign mainly to B–N–H (800–900 cm^–1^, see above); stretching vibration associated with the B–N, N–C, and B–C bonds at 1000–1300 cm^–1^ ; and the C–C stretching vibration in the 1430–1650 cm^–1^ range.^89,90^ Therefore, our calculated frequencies for the stretching vibration of these homo- and heteronuclear bonds are somewhat overestimated, although it should be mentioned that these experimental values are not specific to type-IV BC_2_N nanotubes but to boron–carbonitride nanotubes in general.

### 3.4 Solvation Energy, Dipole Moment, and MEP Isosurface

**Figure 7:**
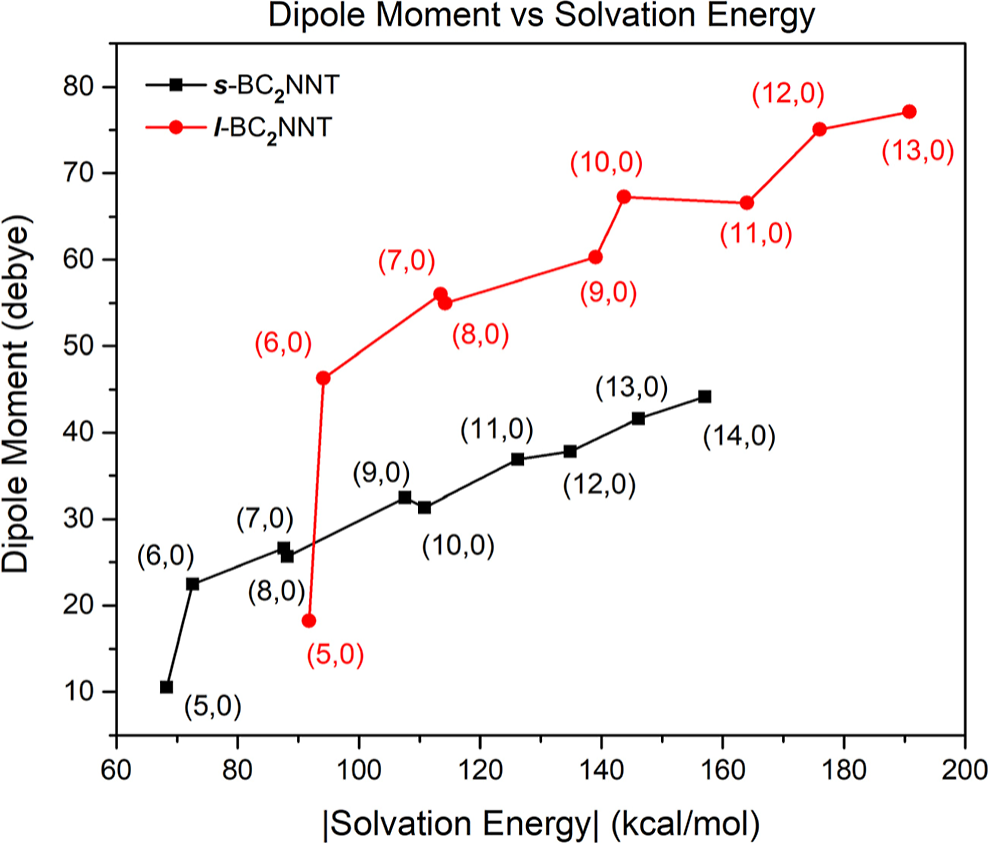
Behavior of the dipole moment (in debyes) in relation to the solvation energy (in kcal/mol). The nanotube corresponding to each point is indicated inside: black squares and red balls for ***s***- and ***l***-BC_2_NNTs, respectively. Values were calculated using the SMD model, as implemented in Gaussian16 software package.

For a better prediction about its possible biological application and its behavior in the aqueous medium, ε = 78.3553, the calculation for the prediction of the solvation energy is performed with SMD, a universal continuous solvation model where its applicability to any charged or uncharged solute in any solvent or liquid medium is denoted.^91^ SMD is generally considered to be more accurate when using high dielectric constants. But it must also be noted that for this work, the SMD model was taken into account due to the consistency when working with the Minnesota functional family, consistent with what was calculated in the present work. The solvation energies (Δ*E*_solv_) were approximated through eq 8
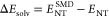
8where *E*_NT_ is the total energy of the optimized nanotube in vacuum, while *E*_NT_^SMD^ is the energy of the nanotube in water solvent calculated using the SMD model. The more negative the Δ*E*_solv_ value, the greater the degree of solubility of the nanotube/water systems. A linear relationship is observed between Δ*E*_solv_ and the chirality index ***n***: a higher ***n*** number is associated with a higher solvation energy value (coefficient of determination, *R*^2^ = 0.988 and 0.979 for ***s***- and ***l***-BC_2_NTs, respectively) (see Figure S8). Although all nanotubes show thermodynamically favorable solvation processes (Δ*E*_solv_ < 0), ***l***-BC_2_NNTs present higher energies (−91.78 to −190.82 kcal/mol), around 30% higher compared to the Δ*E*_solv_ of short nanotubes (−68.28 to −157.08 kcal/mol). These Δ*E*_solv_ values are higher compared to the solvation energies of pristine zigzag-type BNNTs reported previously (−20 to −38 kcal/mol).^86,92^ This suggests that BC_2_NNTs present an advantage in biological applications, where solubility plays a key role (along with biocompatibility). Since solvation processes depend on the intermolecular forces present in a molecular system, and in turn, these forces depend on the polarity of its components, a relationship between Δ*E*_solv_ and the dipole moment (μ⃗) would be expected (see Figure 7). More polar systems have a higher solvation energy.

**Figure 8:**
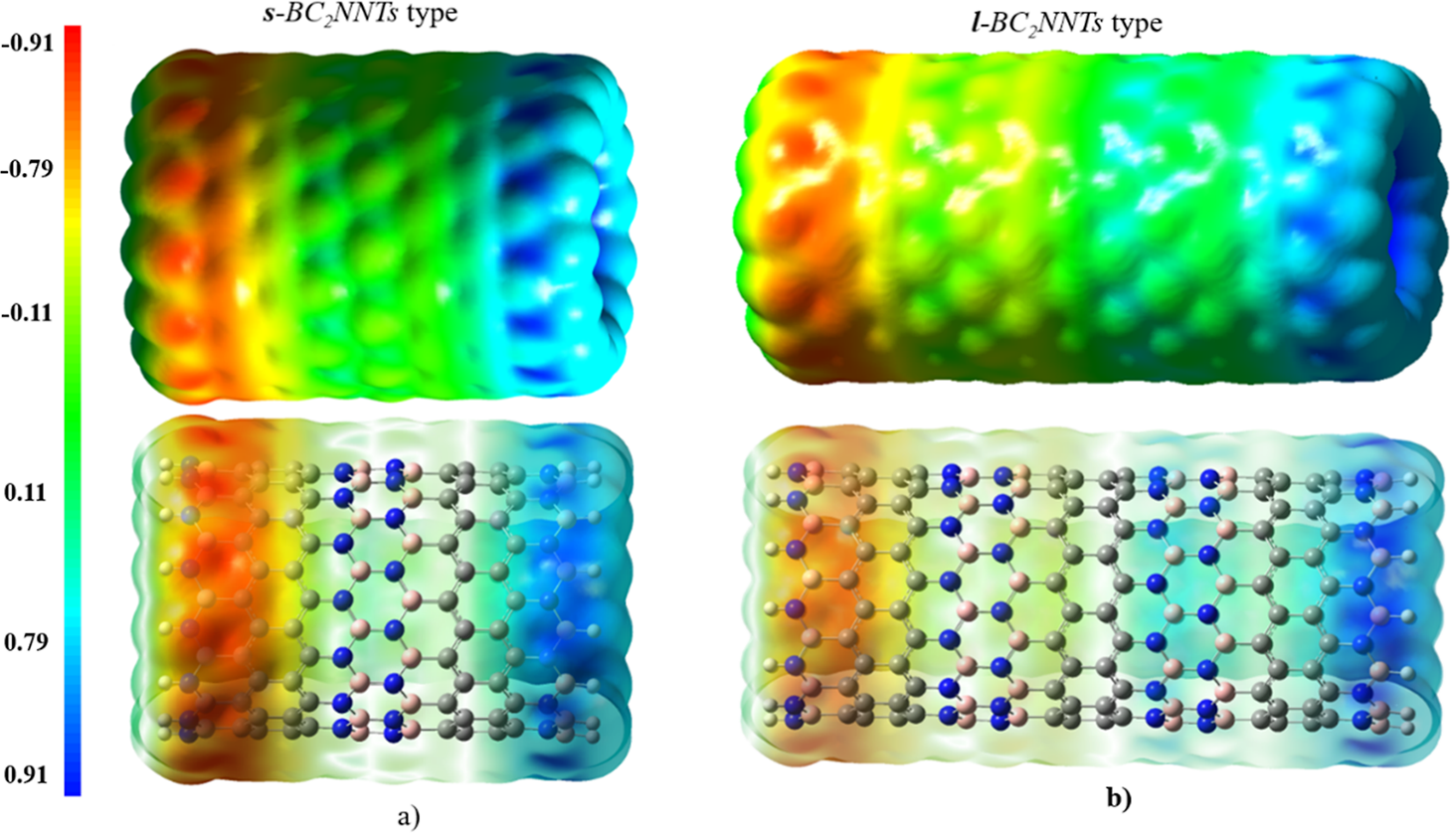
MEP isosurface for the (a) (14,0) ***s***-BC_**2**_NNT and (b) (13,0) ***l***-BC_**2**_NNT, both in side view, solid (top) and transparent (bottom). Red, blue, and green colors represent the negative, positive, and neutral charges, respectively.

Molecular electrostatic potential (MEP) surfaces were calculated for all of the nanotubes studied in this work, generated by mapping the M06-2X/6-31G(d) electrostatic potential. MEP surface plot is a useful tool for the qualitative interpretation of the electrophilic and nucleophilic sites of a molecule through its charge distribution. Figures 8, S9, and S10 show the MEP maps of ***s***- and ***l***-BC_2_NNTs. Electrophilic sites show the most positive electrostatic potential (blue color), while negative regions of electrostatic potential correspond to nucleophilic sites (red color). The zero potential sites are green. A charge polarization is observed in the BC_2_NNTs, where the negative and positive sites are located on the N- and B-side, respectively. At both ends, there is a neutral-to-positive character due to the presence of the dangling hydrogen atoms. Furthermore, the central part of the nanotube shows a neutral character (green color), resulting in a fading of the negative charge as we move from the N-side to the B-side (red–yellow–green–cyan–blue). This charge distribution is different from that observed in pristine zigzag-type BNNTs and CNTs, where the negative charge sites are distributed on the central carbons (in the case of CNTs) or located on the nitrogen atoms along the entire nanotube (in BNNTs).^93−95^ It appears that BC_2_NNTs allow a path for charge transfer leading from the B-side to N-side end, resulting in polarized nanotubes that would favor regioselectivity reactions. Figure S11 shows the Fukui distribution function for (9,0) *l*-BC_2_NNT (as an example, and using the Multiwfn program) to study its local reactivity.^96−98^ According to this, electrophilic attacks (*f*^–^(*r*)) would take place on the N-side of (9,0) *l*-BC_2_NNT, and nucleophilic attacks ((*f*^+^(*r*)) would take place on the B-side. Furthermore, the Fukui distribution function observed in Figure S11 is very similar to that of the MEP isosurface seen in Figure S10.

**Figure 9:**
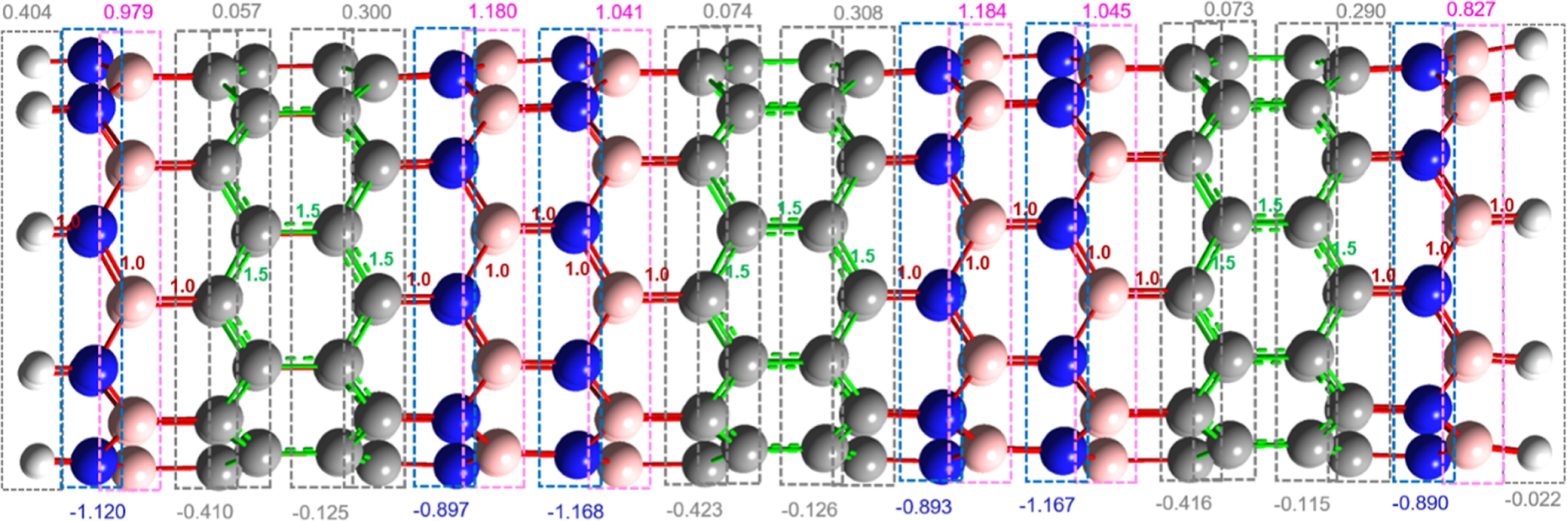
Description of NBO charges and bond order for type-IV (9,0) *l*-BC2N nanotube. (Nitrogen, boron, and carbon atoms in blue, pink, and gray balls, respectively).

Natural Bond Order (NBO) analysis is a method used to study the bond order, interaction parameters, electron configuration, and partial natural charge transfer of molecules.^99^ NBO calculations were performed to describe the natural charge and bond order of the (9,0) *l*-BC_2_N nanotube (Figure 9).

A periodicity of alternating positive and negative NBO charges can be observed along the axial axis of the nanotube. Note that for the system, boron atoms are positive, while nitrogen atoms are negatively charged, while partially, the carbon atoms alternate positive and negative charge. Consequently, rings of positive and negative zones can be observed alternately along the axial axis of the nanotube. On the other hand, the NBO bond order shows that the B–N, B–C, and C–N bonds are of order 1 (single bond; red color), while only the C–C bonds have a value of 1.5 (partial double bond, green color). These results are consistent with those obtained by Mayer’s bond order, discussed in Section 3.1 (Table 2).

## 4 Conclusions

In this work, the structural, vibrational, and electronic properties of zigzag (***n***, 0) BC_2_N nanotubes in their most stable configuration, type IV, were studied through first-principles calculations within the Density Functional Theory and using a M06-2X/6-31G(d) level of theory. We explored the property–structure relationship by focusing on the chirality index (***n***). Furthermore, to analyze the length dependence of the stability/reactivity of BC_2_N nanotubes, short (***n*** = 5–14, ***s***-BC_**2**_NNTs) and long (***n*** = 5–13, ***l***-BC_**2**_NNTs) nanotubes were proposed, with average lengths of 18.07 and 26.74 Å, respectively. Total energy minimization, assuming nonmagnetic nature and charge neutrality, yielded the ground state of all nanostructures. In both sizes, a linear growth of the nanotube diameters with respect to the ***n***-index is observed. The electrophilicity (ω) and nucleophilicity (*N*″) indices show that the BC_2_NNTs are electrophilic systems; however, an increase in the length of the nanotube triples its electrophilic character. The chemical potential (μ) appears to be independent of the chirality index when ***n*** ≥ 6; furthermore, no significant differences were observed in this descriptor between short and long nanotubes. The ***s***-BC_2_NNTs show a semiconductor character, while ***l***-BC_2_NNTs show a semiconductor-to-semimetallic character; therefore, the length of the nanotube is a key element for fine-tuning the conductive properties of these systems. Equations that allow modeling the cohesive energy (*E*_coh_) as a function of the ***n***-index showed that at high ***n*** numbers, a constant value of *E*_coh_ is reached, showing that nanotubes of a larger diameter and length will be favored. Furthermore, a longer axial length of the nanotube improves the solubility properties as it considerably increases the dipole moment (μ⃗) and the solvation energy (Δ*E*_solv_) in water. Finally, BC_2_NNTs showed polarization relative to the distribution of negative and positive charges, as indicated by molecular electrostatic potential maps. This is important for possible regioselective reactions. The set of BC_2_NNTs studied in this work may be proposed for biological applications. Also, due to the molecular gap energy found in the range 0.35 < *E*_g_ < 1.6 eV, we propose that these structures could be applied in the fabrication of integrated circuits at the nanoscale.

## 5 Software and Hardware Details

All calculations were performed with the software Gaussian 16,^52^ Revision B.01, by means of two processors (Intel Xeon E5-2680v3 and 30 M cache, 2.50 GHz, and 24 cores with a total RAM of 512 GB). Optimization was performed via DFT/M06-2*X*/6-31G(d), providing seven-digit precision.

## Supporting Information Available

Examples of the different types of structural models of zigzag-BC2N, total structural parameters of the optimized *s*-BC_2_NNT and *l*-BC_2_NNT nanotubes calculated via DFT/M06-2X/6-31G(d), dipole moment behavior as a function of chirality index (**n**) for zigzag-type *s*-BC_2_NNT and *l*-BC_2_NNT, respectively, total energy behavior as a function of chirality index (**n**) for zigzag-type *s*-BC_2_NNT and *l*-BC_2_NNT, respectively, constitution of the unit cell of *s*-BC_2_NNTs and *l*-BC_2_NNTs and its representation within a nanotube of (5,0) chirality, energy of frontier molecular orbitals (HOMO and LUMO) as a function of the chirality index, the chemical potential behavior as a function of chirality index (**n**) for zigzag-type *s*-BC_2_NNT and *l*-BC_2_NNT, respectively, molecular gap behavior as a function of chirality index (**n**) for zigzag-type *s*-BC_2_NNT and *l*-BC_2_NNT, respectively, solvation energy behavior as a function of chirality index (**n**) for zigzag-type *s*-BC_2_NNT and *l*-BC_2_NNT, respectively, the MEP isosurface for for zigzag-type *s*-BC_2_NNT and *l*-BC_2_NNT, respectively, showing the difference in charge concentration on the opposite plane sides, the Fukui distribution function for (9,0) *l*-BC_2_N nanotube, evidencing the local reactivity: regions susceptible to nucleophilic and electrophilic attacks, and the shaded surface map with projection effect of electron localization function (ELF) of the type-IV (9,0) *l*-BC_2_N nanotube (PDF)
