# Supplementary material for: Quantum Chemical Studies on the Structural, Electronic, and Vibrational Properties of Boron Carbonitride Nanotubes
Source: ACS Omega. 2025 Apr 17;10(16):16031–43. doi: 10.1021/acsomega.4c09158 (PMC12044460; doi:10.1021/acsomega.4c09158)
Supplement: Supplementary file 1 — ao4c09158_si_001.pdf [file ao4c09158_si_001.pdf]

Quantum chemical studies on the structural, electronic and vibrational properties of boron carbonitride nanotubes

Raúl Mendoza-Báez [†], Dolores Garcia-Toral[††], María Teresa Romero de la Cruz[†††], Aracely del Carmen Martínez Olguín [††††], Víctor M. Vázquez-Báez[†††††], Gregorio Hernández Cocoletzi [††††††] and Juan Francisco Rivas-Silva[††††††]*

[†] Departamento de Química, Centro de Investigación y de Estudios Avanzados del IPN (Cinvestav), Av. IPN 2508, Col. San pedro Zacatenco, Ciudad de México 07360, México.

raul.mendoza@cinvestav.mx

[††] Benemérita Universidad Autónoma de Puebla, Facultad de Ingeniería Química, Av. San Claudio y 18 Sur S/N, San Manuel, Puebla 72570, México.

dolores@ifuap.buap.mx

[†††] *Facultad de Ciencias Físico Matemáticas, Universidad Autónoma de Coahuila, Unidad Camporredondo, Edif. A 25000, Saltillo, Coahuila, México*

teresa.romero.cruz@uadec.edu.mx

[††††] *CONAHCyT - Facultad de Ciencias Físico Matemáticas, Universidad Autónoma de Coahuila, Unidad Camporredondo, Edif. A 25000, Saltillo, Coahuila, México.*

aracely.olguin@uadec.edu.mx

[†††††] Facultad de Ingeniería, Benemérita Universidad Autónoma de Puebla, Puebla 72570, Mexico; *manuel.vazquez@correo.buap.mx*

[††††††] Instituto de Física, Benemérita Universidad Autónoma de Puebla, Av. San Claudio y Blvd. 18 Sur, Col. San Manuel, Puebla 72570, Mexico; *cocoletz@ifuap.buap.mx* , *rivas@ifuap.buap.mx*

[*] *Correspondence author: dolores@ifuap.buap.mx*

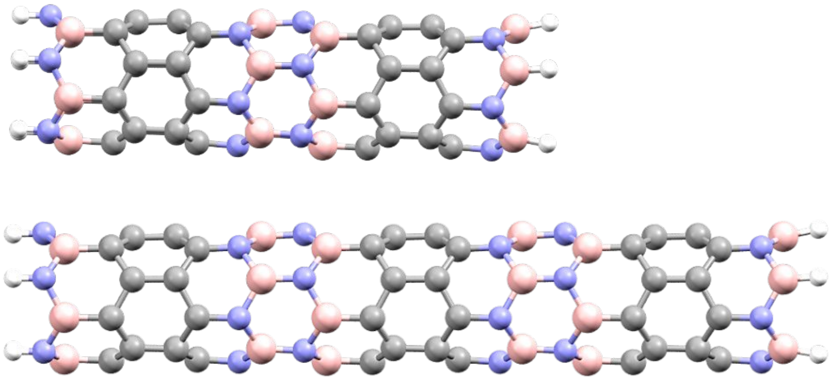

Figure 1S. Two nanotube lengths proposed in this work: short (***s***-BC_2NNT, top) and long (***l***-BC_2NNTs, bottom). In both cases, (5,0) zigzag-type nanotubes were taken as an example. (Nitrogen, boron, carbon and hydrogen atoms in blue, pink, gray and white balls, respectively).

Table 1S. Structural parameters of the optimized *s*-BC_2NNT nanotubes calculated via DFT/M06-2X/6-31G(*d*).

**Nanotube**	**Size (Å)**		**Average Bond Lengths (Å)**					
s-BC_2NNT	Axial length	Diameter	B—C	B—N	C—C	N—C	B—H	N—H
(5,0)	18.018	4.259	1.534	1.466	1.440	1.398	1.180	1.017
(6,0)	18.045	4.976	1.535	1.456	1.433	1.404	1.180	1.017
(7,0)	18.068	5.767	1.535	1.454	1.431	1.404	1.180	1.016
(8,0)	18.082	6.567	1.538	1.451	1.428	1.406	1.181	1.016
(9,0)	18.080	7.336	1.536	1.450	1.427	1.404	1.181	1.015
(10,0)	18.093	8.169	1.538	1.448	1.426	1.406	1.181	1.015
(11,0)	18.089	8.923	1.537	1.447	1.425	1.405	1.181	1.015
(12,0)	18.095	9.779	1.538	1.446	1.425	1.406	1.181	1.015
(13,0)	18.094	10.497	1.538	1.446	1.424	1.406	1.181	1.015
(14,0)	18.096	11.391	1.539	1.445	1.424	1.406	1.181	1.015

Table 2S. Structural parameters of the optimized *l*-BC_2NNT nanotubes calculated via DFT/M06-2X/6-31G(*d*).

**Nanotube**	**Size (Å)**		**Average Bond Lengths (Å)**					
l-BC_2NNT	Axial length	Diameter	B—C	B—N	C—C	N—C	B—H	N—H
(5,0)	26.649	4.240	1.527	1.467	1.441	1.393	1.180	1.017
(6,0)	26.704	4.964	1.531	1.459	1.433	1.401	1.180	1.017
(7,0)	26.732	5.767	1.531	1.456	1.431	1.400	1.180	1.016
(8,0)	26.759	6.556	1.534	1.453	1.428	1.403	1.180	1.016

(9,0)	26.754	7.335	1.532	1.451	1.427	1.401	1.180	1.015
(10,0)	26.773	8.159	1.535	1.449	1.426	1.403	1.181	1.015
(11,0)	26.767	8.921	1.534	1.449	1.426	1.402	1.181	1.015
(12,0)	26.776	9.764	1.535	1.448	1.425	1.403	1.181	1.015
(13,0)	26.776	10.497	1.535	1.447	1.425	1.403	1.181	1.015

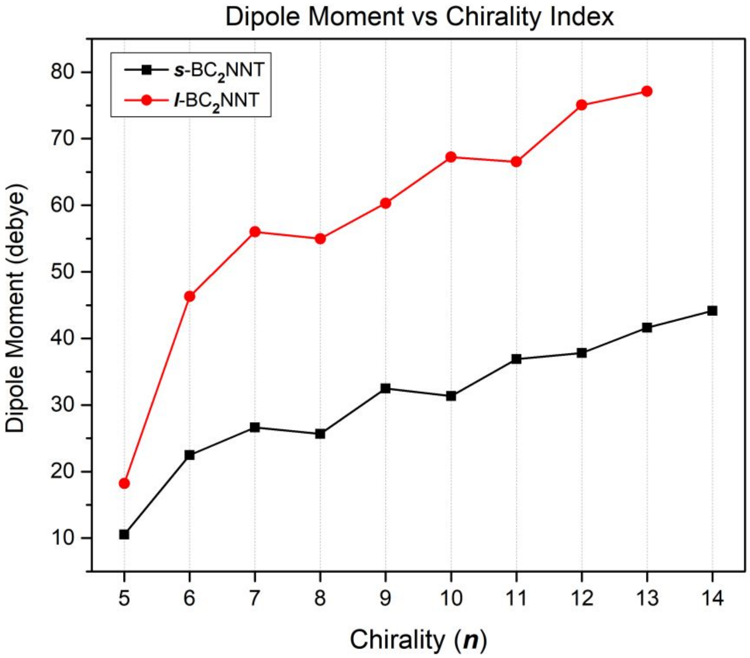

Figure 2S. Dipole moment (Debye) behavior as a function of chirality index (***n***). Solid black and red lines for zigzag-type *s*-BC_2NNTs and *l*-BC_2NNTs, respectively.

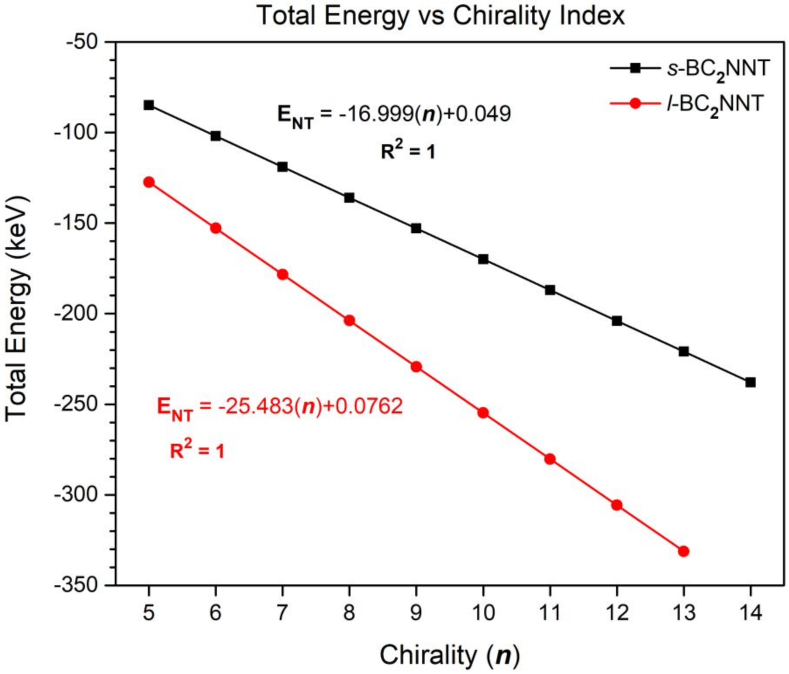

Figure 3S. Total energy (in keV) behavior as a function of chirality index (***n***). Solid black and red lines for zigzag-type *s*-BC_2NNTs and *l*-BC_2NNTs, respectively. Inside, the linear fit equations.

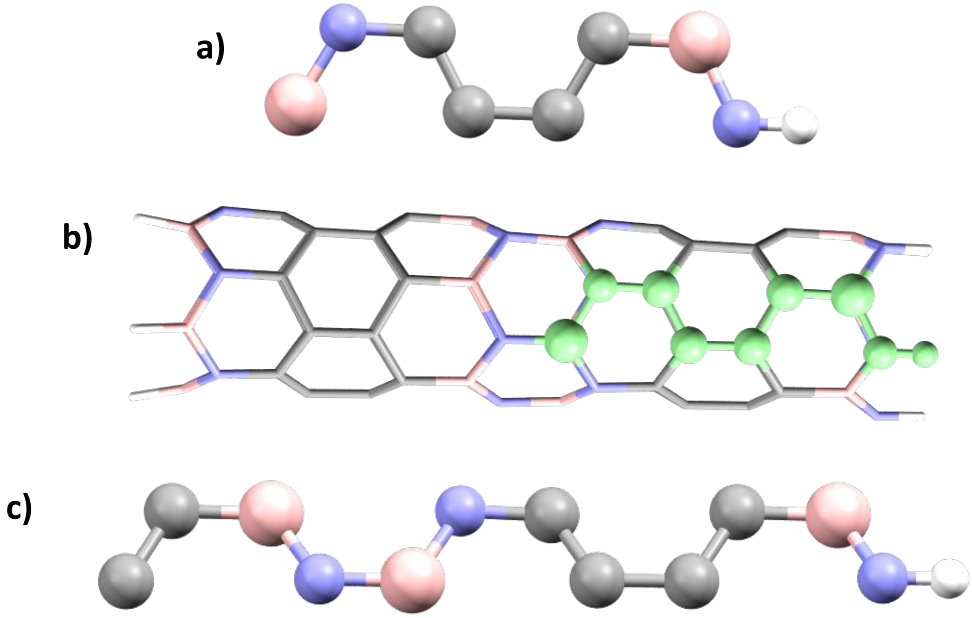

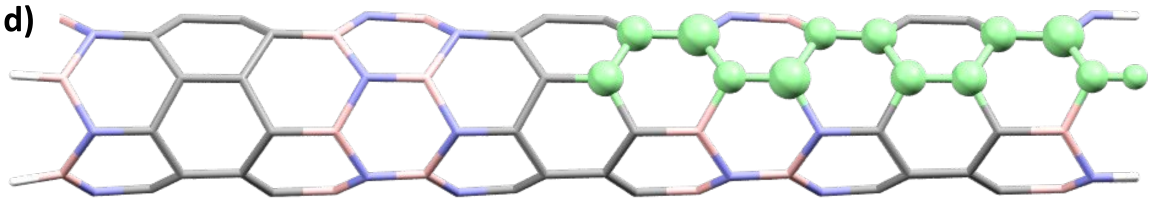

Figure 4S. Unit cell configuration for (a) ***s***-BC_2NNTs and (c) ***l***-BC_2NNTs, and their representation within a (5,0) nanotube highlighted in green ((b) and (d), respectively).

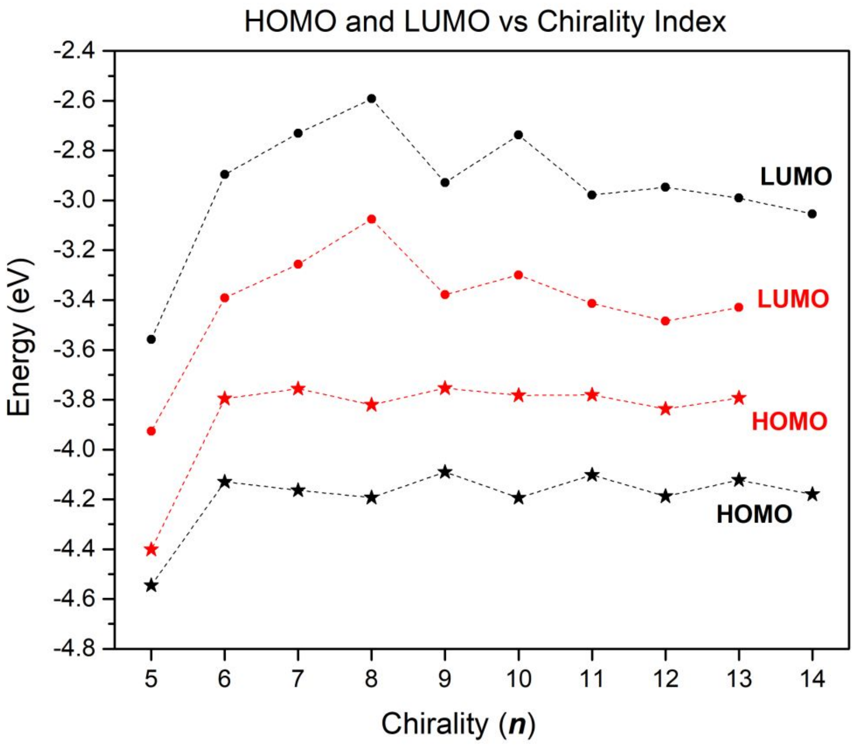

Figure 5S. Energy of frontier molecular orbitals (HOMO and LUMO) as a function of the chirality index (***n***). Black and red dashed lines for ***s***- and ***l***-BC_2NNTs, respectively. Solid balls correspond to LUMO and stars to HOMO.

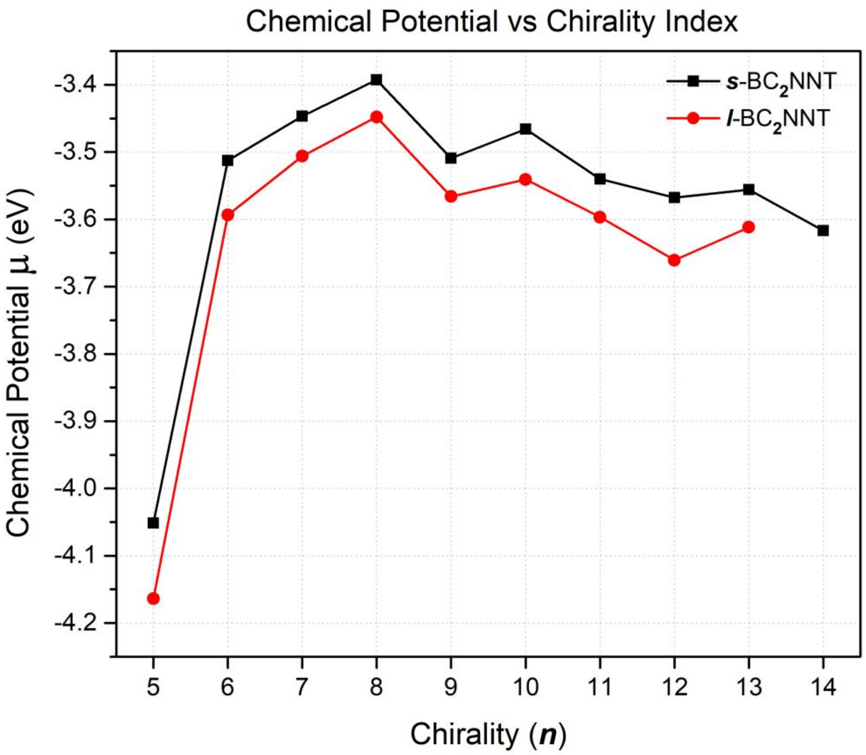

Figure 6S. Chemical potential (μ, in eV) behavior as a function of chirality index (***n***). Solid black and red lines for zigzag-type *s*-BC_2NNTs and *l*-BC_2NNTs, respectively.

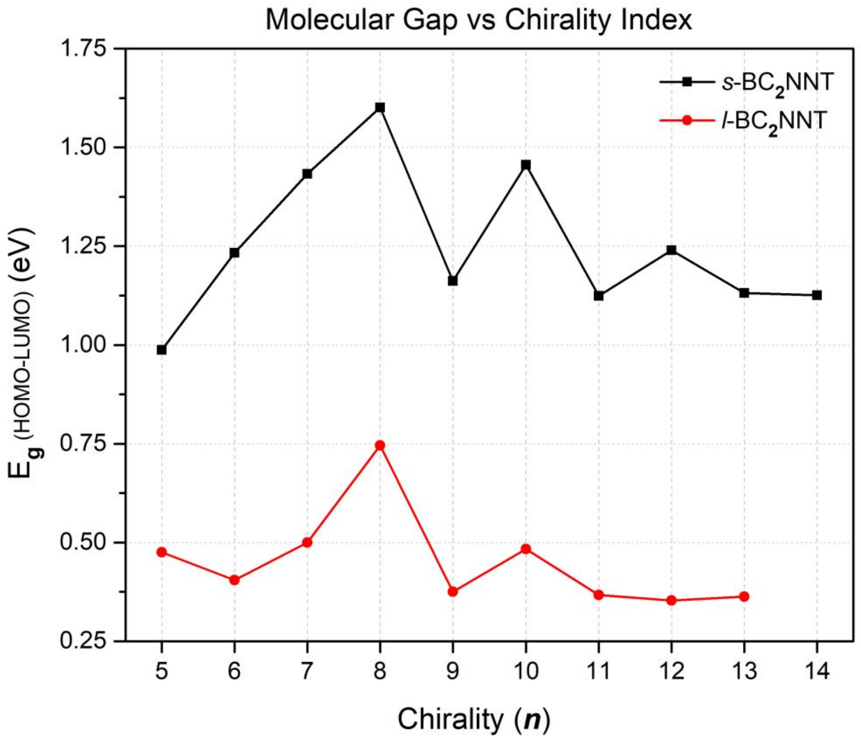

Figure 7S. Molecular Gap (E_g, in eV) behavior as a function of chirality index ($\boldsymbol{n}$). Solid black and red lines for zigzag-type *s*-BC_2NNTs and *l*-BC_2NNTs, respectively

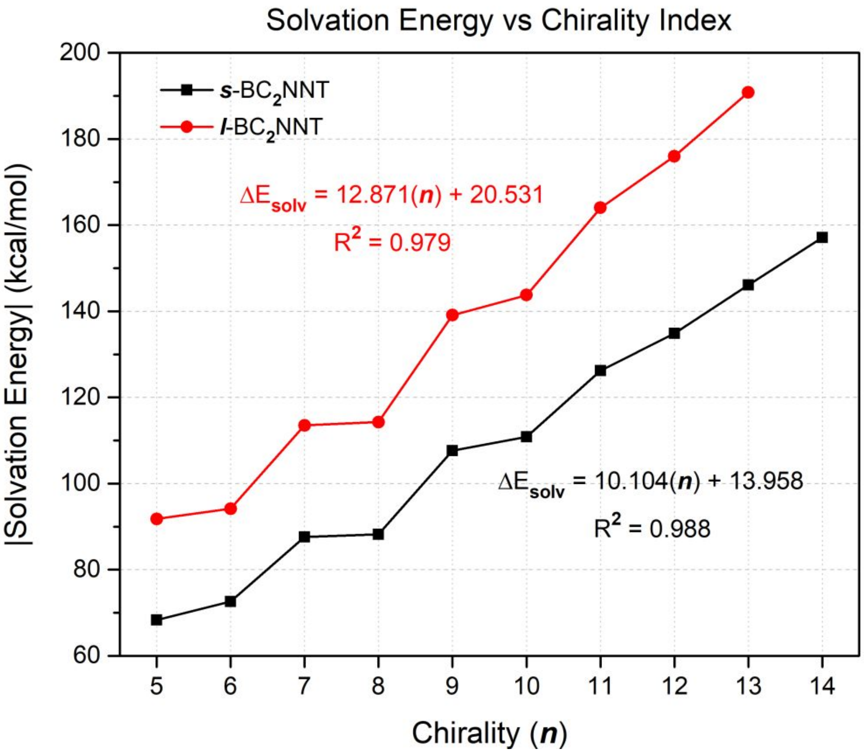

Figure 8S. Solvation energy (ΔE_{solv}, in kcal/mol) behavior as a function of chirality index ($\boldsymbol{n}$). Solid black and red lines for zigzag-type *s*-BC_2NNTs and *l*-BC_2NNTs, respectively. Inside, the linear fit equations.

MEP Isosurface ***s***-*BC_2NNTs* type						
**Chirality**	**Front View Solid Negative**	**Front View Transparent Negative**	**Side View Transparent**	**Side View Solid**	**Front View Transparent Positive**	**Front View Solid Positive**
**(5,0)**	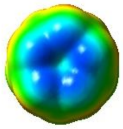	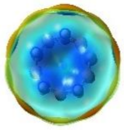	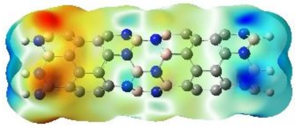	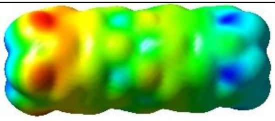	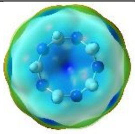	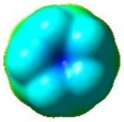
**(6,0)**	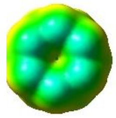	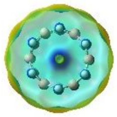	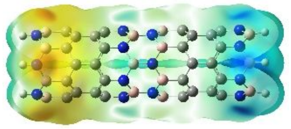	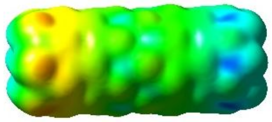	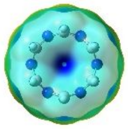	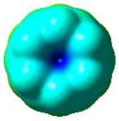

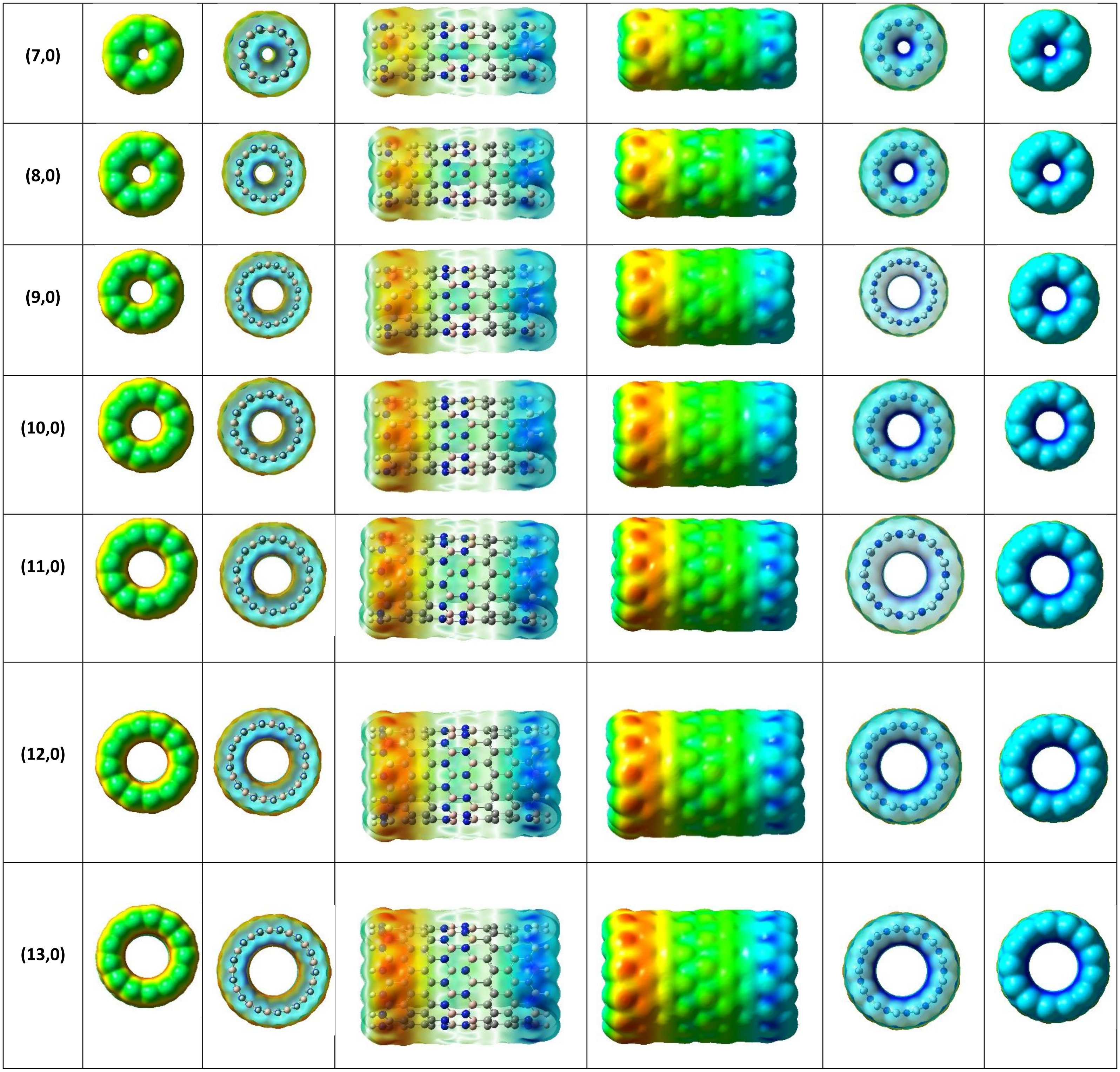
(7,0)
(8,0)
(9,0)
(10,0)
(11,0)
(12,0)
(13,0)

**(14,0)**	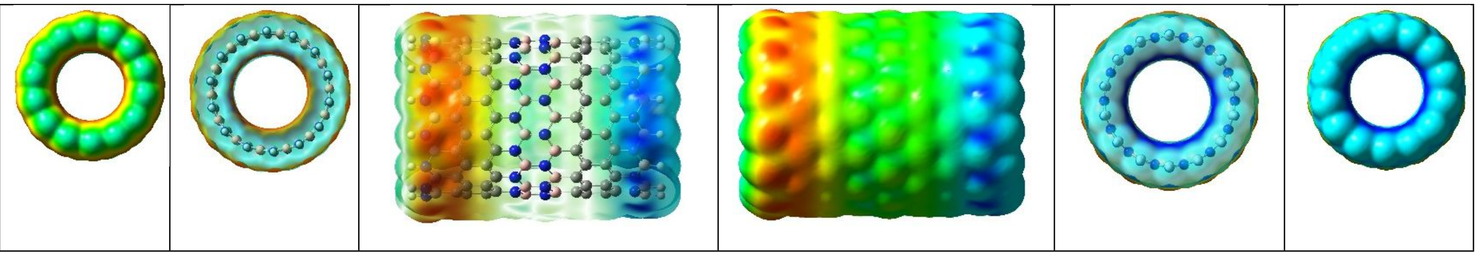					

Figure 9S. MEP isosurface for the **s**-BC_2N nanotubes showing the difference in charge concentration on the opposite plane sides. Red represents the negative charge, blue the positive charge, and green the neutral charge.

MEP Isosurface ***l**-BC_2NNTs* type						
**Chirality**	**Front View Solid Negative**	**Front View Transparent Negative**	**Side View Transparent**	**Side View Solid**	**Front View Transparent Positive**	**Front View Solid Positive**
**(5,0)**	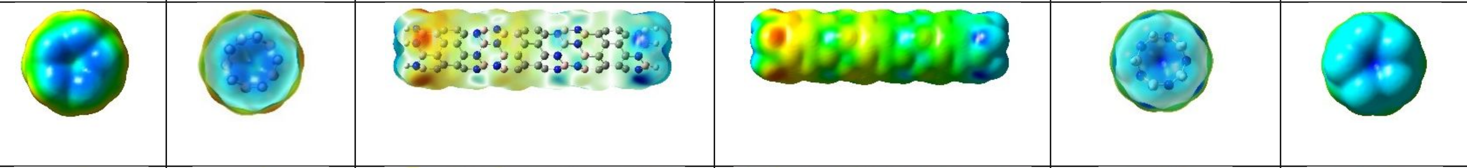					
**(6,0)**	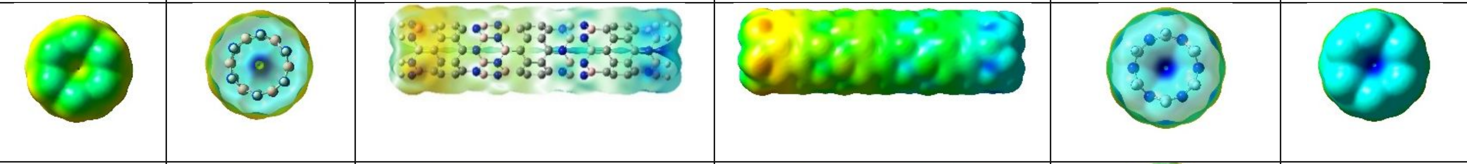					
**(7,0)**	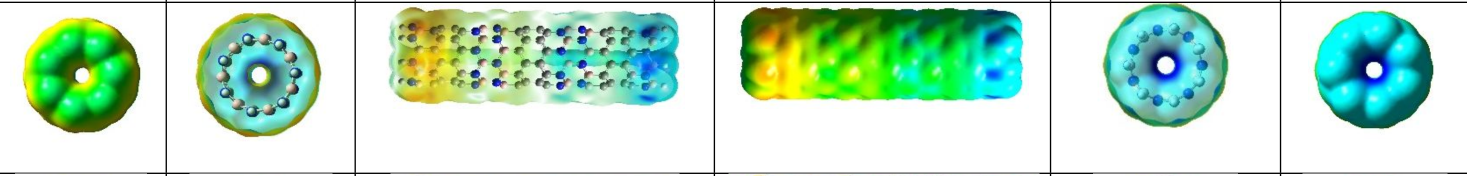					
**(8,0)**	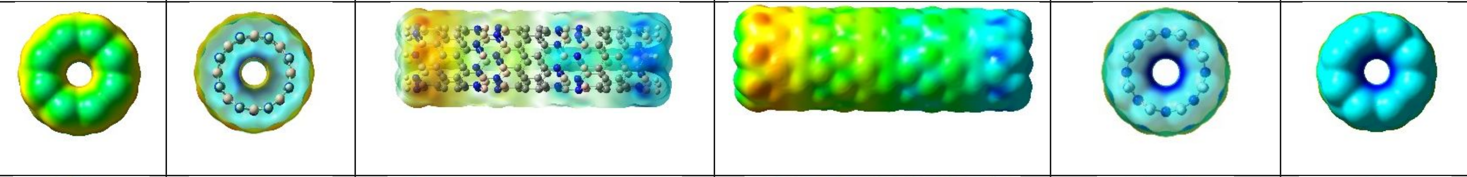					
**(9,0)**	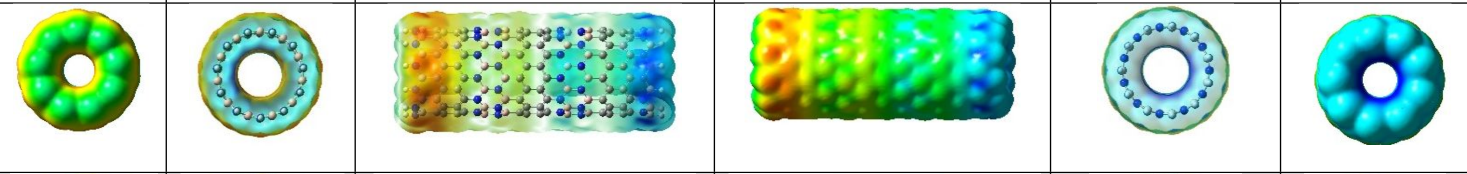					
**(10,0)**	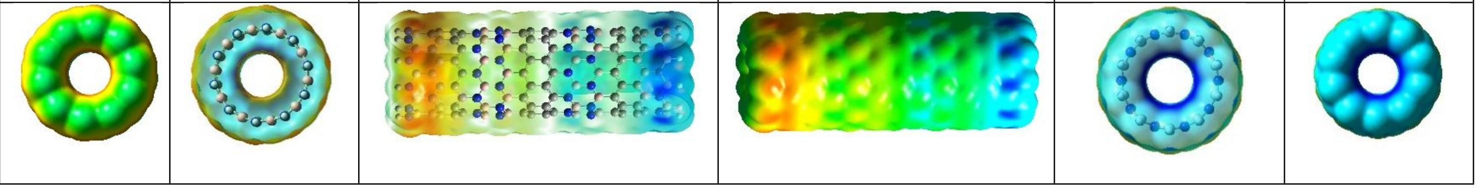					

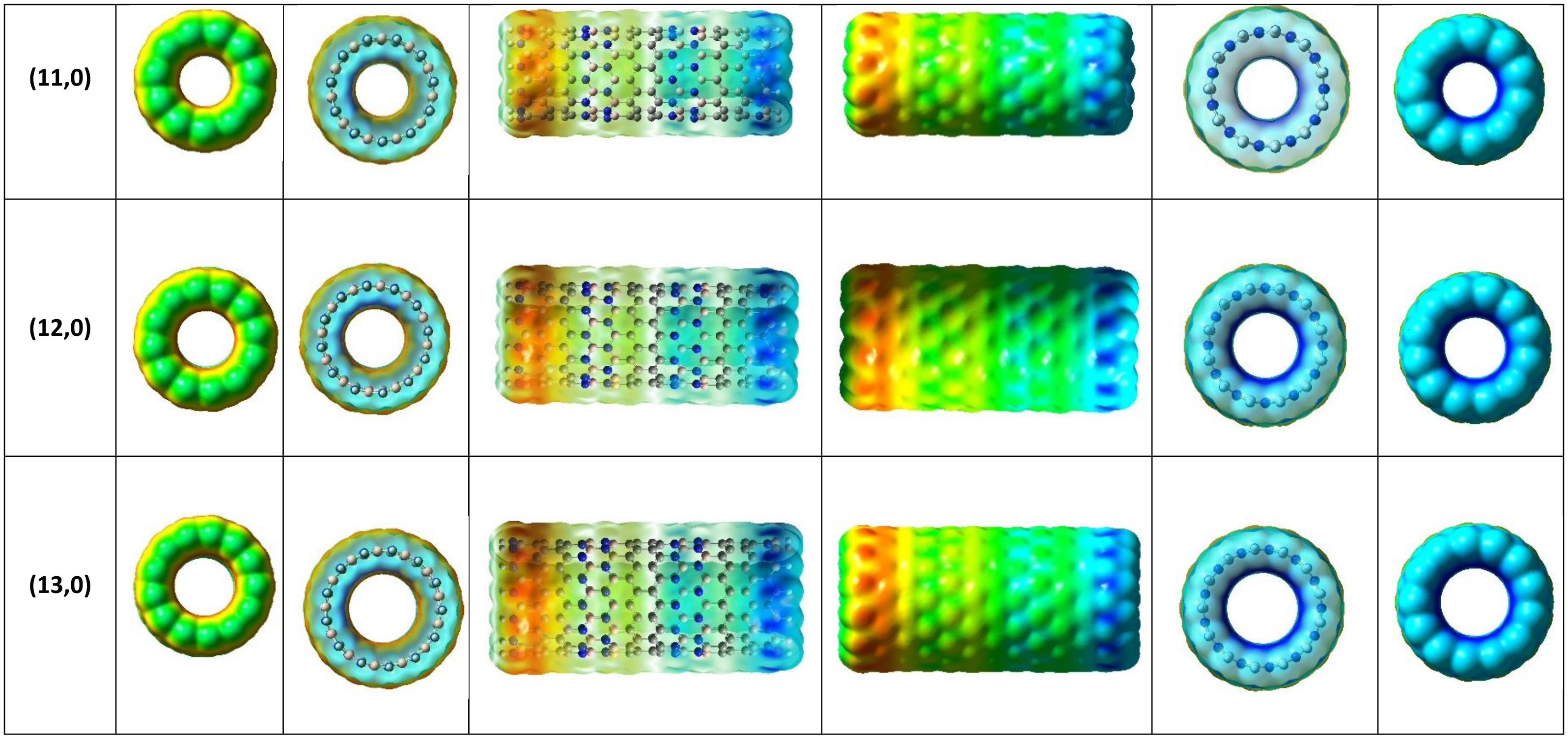

Figure 10S. MEP isosurface for the *l*-BC_2N nanotubes showing the difference in charge concentration on the opposite plane sides. Red represents the negative charge, blue the positive charge, and green the neutral charge.

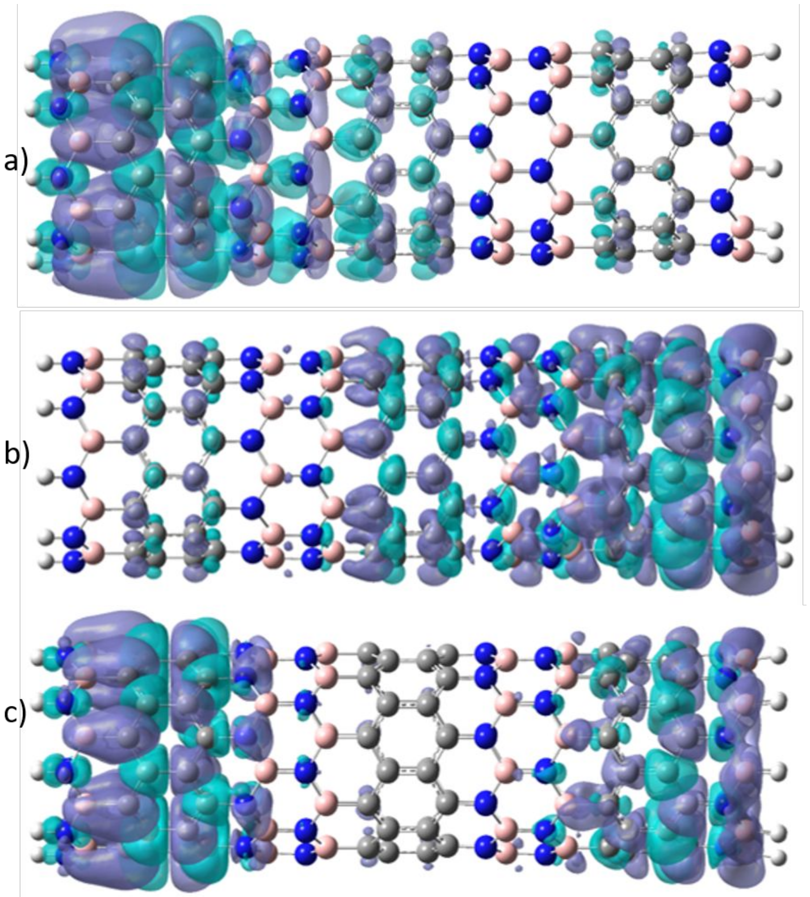

Figure 11S. Fukui function a) Electrophilic attack for $f^{-}(r)$, b) Nucleophilic attack for $f^{+}(r)$ and c) Radical attack for $f^{0}(r)$ for the finite (9,0) *l*-BC_2N nanotube Type IV, calculated with the Multiwfn program.[97, 98]

The following figure shows the isosurface representation of the (Electron Localization Function) ELF for the example of the (9,0) *l*-BC2N nanotube Type IV. Can see that C-C covalent bond regions have high LOL (Localized Orbital Locator) value, followed by the B-N, N-C and B-C bonds, rendering high degree of electron localization in that places. According to the color scale, where red has the maximum value and navy blue the minimum value.

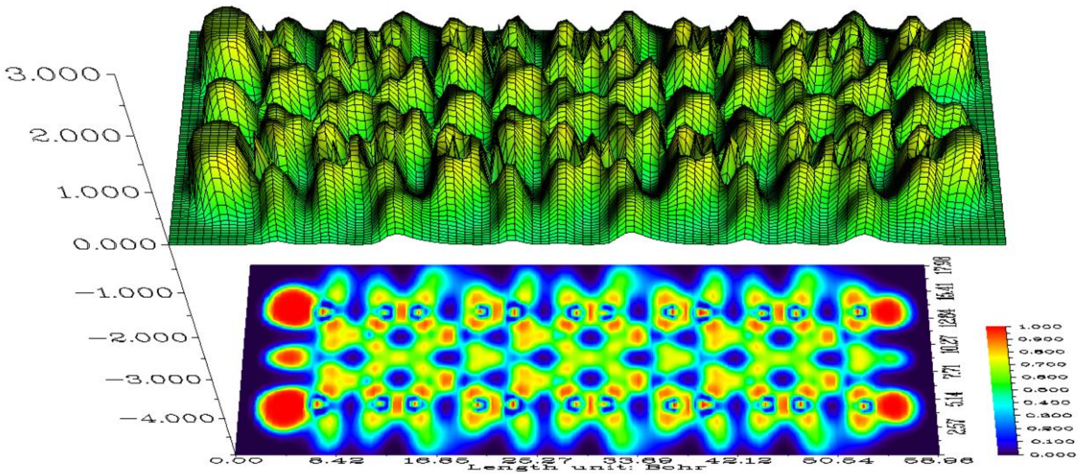

Figure 12S. Shows the shaded surface map with projection effect of electron localization function (ELF) [98] of the type-IV (9,0) *l*-BC2N nanotube.
